# Amino acid residues at core protein dimer-dimer interface modulate multiple steps of hepatitis B virus replication and HBeAg biogenesis

**DOI:** 10.1371/journal.ppat.1010057

**Published:** 2021-11-09

**Authors:** Hui Liu, Junjun Cheng, Usha Viswanathan, Jinhong Chang, Fengmin Lu, Ju-Tao Guo

**Affiliations:** 1 Department of Microbiology & Infectious Disease Center, School of Basic Medical Sciences, Peking University Health Science Center, Beijing, China; 2 Baruch S. Blumberg Institute, Doylestown, Pennsylvania, United States of America; The Pennsylvania State University College of Medicine, UNITED STATES

## Abstract

The core protein (Cp) of hepatitis B virus (HBV) assembles pregenomic RNA (pgRNA) and viral DNA polymerase to form nucleocapsids where the reverse transcriptional viral DNA replication takes place. Core protein allosteric modulators (CpAMs) inhibit HBV replication by binding to a hydrophobic “HAP” pocket at Cp dimer-dimer interfaces to misdirect the assembly of Cp dimers into aberrant or morphologically “normal” capsids devoid of pgRNA. We report herein that a panel of CpAM-resistant Cp with single amino acid substitution of residues at the dimer-dimer interface not only disrupted pgRNA packaging, but also compromised nucleocapsid envelopment, virion infectivity and covalently closed circular (ccc) DNA biosynthesis. Interestingly, these mutations also significantly reduced the secretion of HBeAg. Biochemical analysis revealed that the CpAM-resistant mutations in the context of precore protein (p25) did not affect the levels of p22 produced by signal peptidase removal of N-terminal 19 amino acid residues, but significantly reduced p17, which is produced by furin cleavage of C-terminal arginine-rich domain of p22 and secreted as HBeAg. Interestingly, p22 existed as both unphosphorylated and phosphorylated forms. While the unphosphorylated p22 is in the membranous secretary organelles and the precursor of HBeAg, p22 in the cytosol and nuclei is hyperphosphorylated at the C-terminal arginine-rich domain and interacts with Cp to disrupt capsid assembly and viral DNA replication. The results thus indicate that in addition to nucleocapsid assembly, interaction of Cp at dimer-dimer interface also plays important roles in the production and infectivity of progeny virions through modulation of nucleocapsid envelopment and uncoating. Similar interaction at reduced p17 dimer-dimer interface appears to be important for its metabolic stability and sensitivity to CpAM suppression of HBeAg secretion.

## Author summary

Binding of CpAMs at a HAP pocket between Cp dimer-dimer interfaces misdirects HBV capsid assembly. We demonstrated herein that CpAM-resistant mutations with single amino acid substitution of Cp residues at the HAP pocket not only confer resistance to CpAM suppression of pgRNA packaging, but also to the induction of capsid structure alteration and mature nucleocapsid uncoating as well as the inhibition of cccDNA synthesis by GLS4, a type I CpAM currently in phase 2 clinical trials. Moreover, these mutations in the context of precore protein also conferred resistance to CpAM inhibition of HBeAg secretion. We have thus obtained genetic evidence suggesting that CpAMs disrupt the multiple steps of HBV replication and inhibit HBeAg secretion by misdirecting the interaction of Cp or reduced p17 dimers at the HAP pocket. The mechanistic insights gained from this study should facilitate the development of Cp/p17-targeting antiviral drugs for the treatment of chronic hepatitis B.

## Introduction

Hepatitis B virus (HBV) chronically infects 257 million people worldwide and causes more than 800,000 deaths annually, due to cirrhosis, hepatocellular carcinoma and liver failure [1]. Although the long term suppression of viral replication by nucleos(t)ide analogue viral DNA polymerase inhibitors reduces the risk of death due to liver diseases by 50–70% [2–4], less than 5% of those treated patients achieve the loss of circulating HBV surface antigen (HBsAg), *i*.*e*., the functional cure of chronic hepatitis B (CHB) [5,6]. Novel therapeutics for more potent suppression of viral replication and reinvigoration of host innate and adaptive antiviral immune responses are under preclinical or clinical development [7]. Thus far, at least eight HBV core protein allosteric modulators (CpAMs) have been evaluated in clinical trials and demonstrated to reduce viral load by 1.4 to 3.3 log10 in 28-day proof-of-concept studies in treatment-naïve CHB patients [8]. Combination therapy of CpAM (ABI-H0731) and DNA polymerase inhibitor (entecavir) demonstrated faster and greater declines in serum HBV DNA and RNA following 24 weeks of administration [9].

HBV core protein (Cp) is a 183-amino acid (aa) polypeptide containing a N-terminal assembly domain (NTD, aa 1–140) and an arginine-rich C-terminal domain (CTD, aa150-183), which are connected by a linker of 9 amino acid residues (illustrated in S1 Fig). Cp can self-assemble into empty capsids or package viral DNA polymerase (Pol) and pregenomic RNA (pgRNA) to form nucleocapsids where the reverse transcriptional replication of viral DNA takes place [10]. As the building block of nucleocapsids, Cp also plays essential roles in virion production/infectivity, viral genomic DNA uncoating and transportation into the nucleus of infected hepatocyte for covalently closed circular DNA (cccDNA) synthesis [11–14]. In addition to Cp, a precore protein (pre-C), the precursor of secreted HBV e antigen (HBeAg), is translated from pre-C mRNA and contains an additional 29 amino acid residues at the N-terminus of Cp (illustrated in S1 Fig). The N-terminal 19 aa of pre-C is a signal peptide that targets the nascent pre-C (p25) to the endoplasmic reticulum (ER). Cleavage of the signal peptide by signal peptidase in the ER produces p22, which is subsequently transported to the Golgi complex where the C-terminal arginine-rich domain is cleaved by furins to yield p17 [15,16]. The p17 is subsequently secreted out of hepatocytes as soluble homodimers, designated as HBeAg [17,18].

CpAMs had been shown to misdirect the assembly of capsids by binding to a hydrophobic HAP pocket between the Cp dimer-dimer interfaces [19–21]. Depending on their chemical structure, binding pose and unique interaction with Cp residues at the HAP pocket, CpAMs induce the assembly of either aberrant Cp polymers or morphologically normal capsids devoid of Pol-pgRNA complex, which precludes the viral DNA replication [8]. In addition, it had also been demonstrated that CpAMs induce mature nucleocapsid disassembly and inhibit *de novo* cccDNA synthesis and production of HBeAg [22,23], usually at concentrations that are approximately 10 and 500 fold higher than that required to inhibit viral DNA replication in hepatocytes, respectively [24,25]. Thus far, it remains to know whether these additional pharmacological effects of CpAMs are also resulted from their interaction with Cp or precore-derived proteins at the HAP pocket. Recently, we identified a panel of mutant Cp with a single amino acid substitution of residues at the wall of the HAP pocket conferred resistance to CpAM suppression of pgRNA packaging [26]. Herein, we showed that these CpAM-resistant mutations also conferred resistance to the induction of mature nucleocapsid disassembly as well as inhibition of cccDNA synthesis and HBeAg secretion in hepatocytes by GLS4, a type I CpAM currently in phase 2 clinical trials with the brand name Morphothiadin [27,28]. We have thus obtained genetic evidence supporting the hypothesis that CpAMs not only modulate the interaction of Cp dimers at the “HAP” pocket to misdirect their assembly, but also bind to the “HAP” pocket in the context of assembled capsids to alter their global structures and trigger the disassembly of mature nucleocapsids to modulate cccDNA biosynthesis [22]. Moreover, our studies also revealed important functions of amino acid residues at the Cp dimer-dimer interface in multiple steps of HBV replication and particularly, in HBeAg biogenesis. We obtained evidence suggesting that CpAM inhibition of HBeAg is most likely by targeting reduced p17 dimers for aberrant assembly, but not p22 [29]. The mechanistic insights on the metabolism and functions of Cp and precore-derived proteins in HBV infection of hepatocytes gained from this study should facilitate the discovery and development of novel CpAMs that not only potently inhibit HBV replication, but also more efficiently suppress the biogenesis of cccDNA and HBeAg to achieve the functional cure of CHB.

## Result

### Cp mutations conferring resistance to CpAM inhibition of pgRNA packaging also confer resistance to GLS4-induced capsid structure alterations

**Fig 1:**
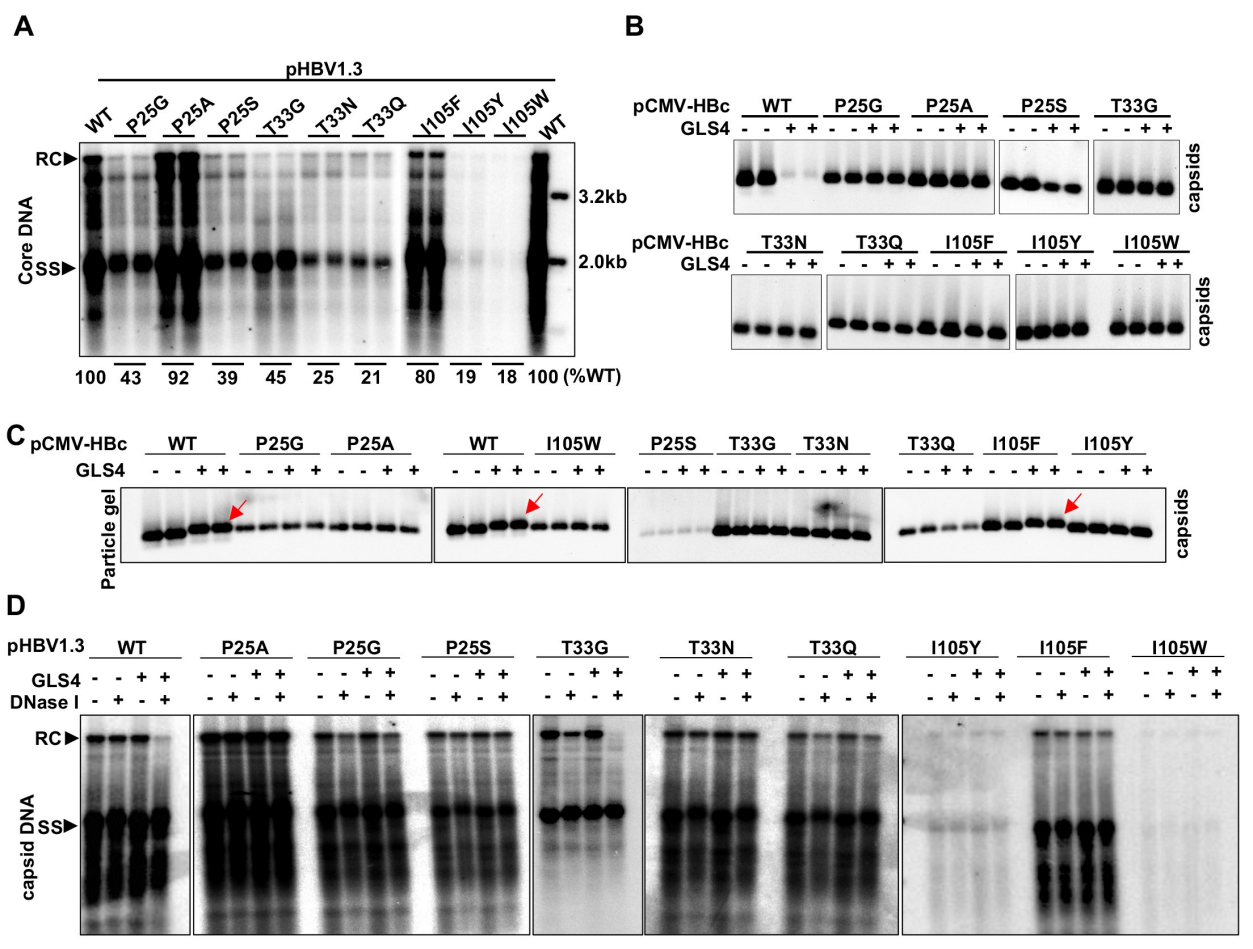
Substitution of Cp residue P25, T33 or I105 confers resistance to GLS4 modulation of capsid assembly and induction of structural alterations of assembled capsids. **(A)** HepG2 cells were transfected with pHBV1.3 or derived plasmid encoding Cp with the indicated single amino acid substitution and harvested at 72 h post transfection. Intracellular core DNA were extracted and analyzed by Southern blot hybridization with an α-^32^P-UTP labeled full-length positive-strand HBV RNA probe. **(B)** HepG2 cells cultured in 12 well plates were transfected with pCMV-HBc or derived plasmid encoding Cp with the indicated single amino acid substitution. At 6 h post transfection, the cells were mock (DMSO)-treated or treated with 0.5 μM GLS4 for 48 h. Intracellular capsids were determined by particle gel assay. **(C)** HepG2 cells were transfected with pCMV-HBc and derived plasmid encoding Cp with the indicated single amino acid substitution harvested at 72 h post transfection. Capsids purified from the cytoplasmic lysates were incubated in a buffer containing 2 μM GLS4 or control solvent (DMSO) at 37°C for 6 h. The electrophoresis mobility of capsids was determined by particle gel assay. **(D)** HepG2 cells were transfected with pHBV1.3 or derived plasmid encoding Cp with the indicated single amino acid substitution and harvested at 72 h post transfection. Capsids purified from the cytoplasmic lysates were incubated in 1× endogenous DNA polymerase reaction buffer in the absence or presence of 1μM GLS4 at 37°C for 16 h. DNA were extracted without or with prior DNase I digestion at 37°C for 30 min. HBV DNA replication intermediates were detected by Southern blot hybridization with an α-^32^P-UTP labeled full-length positive-strand HBV RNA probe. RC, relaxed circular DNA. SS, single stranded DNA. The gray values of HBV DNA in Southern blot assay (panel A) were quantified by Image J and presented as the percentage of that in cells transfected with WT HBV replicon.

In an effort to identify Cp amino acid residues critical for CpAM misdirection of capsid assembly, we performed single amino acid substitution analysis of 25 amino acid residues around the HAP pocket and identified that substitution of Cp residue P25, T33 or I105 by multiple amino acid residues confer resistance to the inhibition of pgRNA packaging and viral DNA replication by several chemotypes of CpAMs [26]. As shown in Fig 1A, the plasmids containing 1.3mer HBV genome encoding wild-type (WT) or mutant Cp with P25G, P25A, P25S, T33G, T33N, T33Q, I105F, I105Y or I105W substitution supported variable levels of HBV DNA replication in HepG2 cells. The significantly reduced replication of HBV DNA with I105Y or I105W mutant Cp was apparently due to the reduced encapsidation of pgRNA (S2 Fig) [26]. In agreement with our previous report [26], all the nine mutant Cp conferred strong resistance to GLS4 inhibition of capsid assembly (Fig 1B) and HBV DNA synthesis in HepG2 cells (S1 Table).

In addition to modulating capsid assembly, we reported previously that GLS4 treatment also selectively induced the disassembly of nucleocapsids containing relaxed circular DNA (rcDNA), but not single-stranded DNA (ssDNA) [22,30]. Moreover, it was reported recently that treatment of purified HBV capsids with other HAP compounds induced the electrophoresis mobility shift of capsids [24,31], an indication of the global structure alteration of capsids [32]. In order to investigate whether the effects of CpAMs on the assembled capsids and mature nucleocapsids also depend on their specific interaction with key amino acid residues at the HAP pocket between Cp inter-dimer interface, we examined if the CpAM-resistant Cp mutations also confer resistance to GLS4 induction of capsid mobility shift and mature nucleocapsid uncoating. To this end, capsids purified from HepG2 cells transfected with a plasmid expressing WT or the indicated CpAM-resistant Cp were incubated *in vitro* without or with GLS4 and then subjected to native agarose gel electrophoresis and immunoblotting detection. In agreement with the previous report [24], GLS4 treatment reduced the electrophoresis mobility of WT capsids. However, GLS4 treatment did not apparently alter the mobility of capsids assembled from CpAM-resistant Cp, except for I105F Cp, which migrated slightly slower upon GLS4 treatment (Fig 1C). To examine the effect of GLS4 on nucleocapsids, capsids purified from the cytoplasmic lysates of HepG2 cells transfected with plasmids containing 1.3mer HBV genome encoding WT or the indicated CpAM-resistant Cp were incubated in endogenous DNA polymerase reaction buffer in the absence or presence of 1 μM GLS4 at 37°C for 16 h. HBV DNA were extracted without or with prior DNase I digestion. HBV DNA in the intact nucleocapsids are protected from DNase I digestion [33], whereas the disassembly or dramatic structure alterations of nucleocapsids releases or exposes HBV DNA for DNase I digestion [22]. Consistent with our previous report [22], Southern blot analyses of HBV DNA replication intermediates indicated that GLS4 treatment of nucleocapsids purified from cells replicating WT HBV rendered the susceptibility of rcDNA, but not other viral DNA species, such as single stranded and incomplete double-stranded DNA, to DNase I digestion. This result implies that GLS4 treatment selectively induce the disassembly of mature nucleocapsids [22]. However, the rcDNA in the nucleocapsids assembled from CpAM-resistant Cp, except for T33G, were resistant to GLS4 induced DNase I digestion (Fig 1D). Interestingly, the fact that rcDNA species in Cp P25G and T33G capsids were partially sensitive to DNase I digestion without GLS4 treatment implies that these mutations partially destabilize mature nucleocapsids under this experimental condition.

Taking together, the results presented above strongly support the notion that similar to its modulation of capsid assembly, GLS4 interacts with the same key amino acid residues at the HAP pocket between Cp inter-dimer interface to induce structure alteration of capsids and promote the disassembly of mature nucleocapsids.

### CpAM-resistant Cp mutations reduce virion production and infectivity and confer resistance to GLS4 inhibition of *de novo* cccDNA synthesis

**Fig 2:**
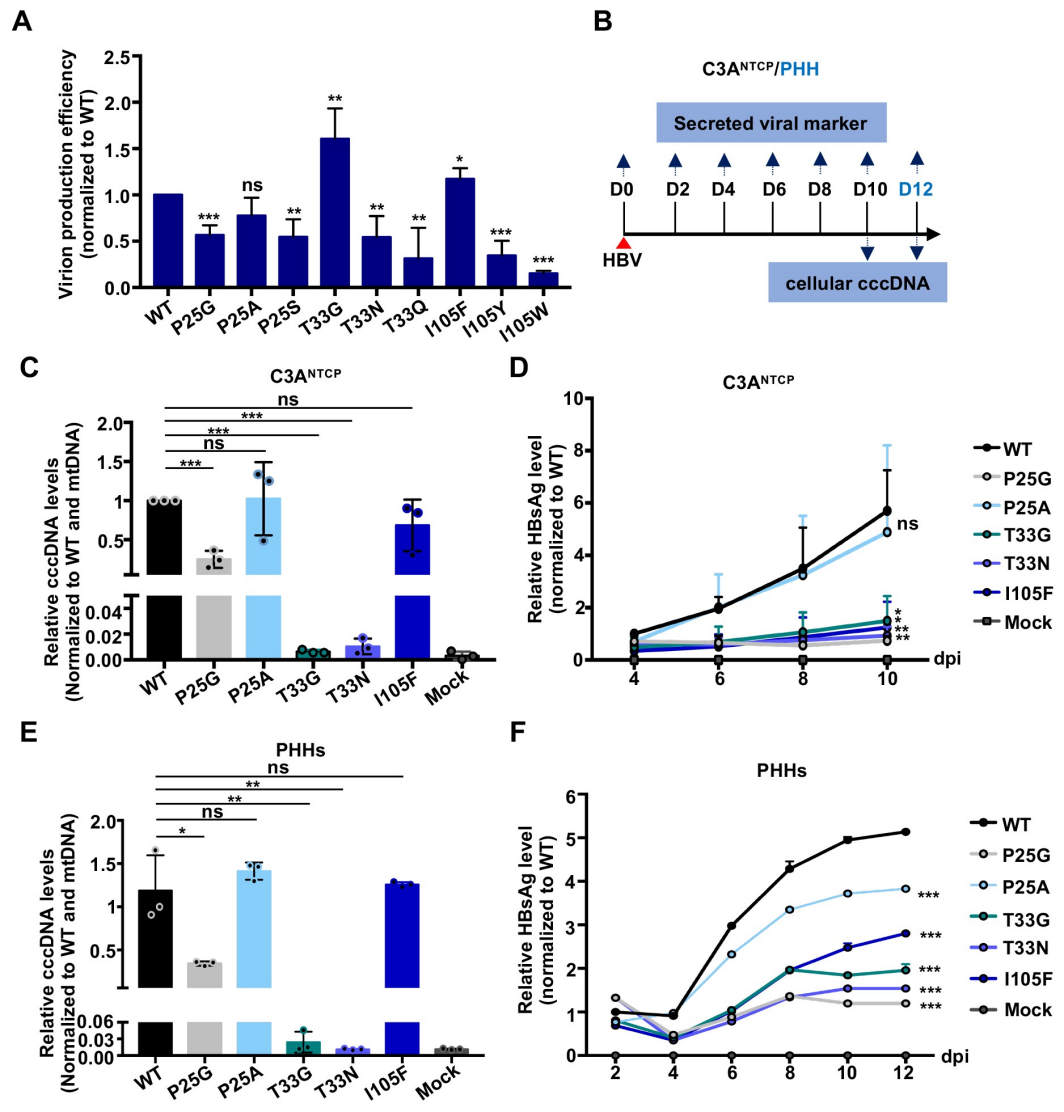
Substitution of Cp residues P25, T33 or I105 reduces the yield and infectivity of virions. **(A)** HepG2 cells were transfected with pHBV1.3 or a derived plasmid encoding Cp with the indicated single amino acid substitution and harvested at 72 h post transfection. Virions in culture media were immunoprecipitated with antibodies recognizing epitopes in S and pre-S2 regions of envelope proteins and virion DNA was quantified by qPCR (IP-qPCR assay). Intracellular core DNA was quantified by qPCR assay. The virion production efficiency of HBV replicons was calculated by dividing the virions yield with the level of intracellular HBV DNA of the corresponding replicon after normalization to the values from cells transfected with WT HBV replicon. Results (Mean ± SD) from at least three independent experiments are presented. **(B)** Schematic presentation of HBV infection experimental schedule. (**C** and **D**) C3A^NTCP^ cells were infected with WT or Cp mutant HBV at a multiplicity of infection (MOI) of 500 genome equivalent (GEq). Culture medium was changed every other day. The cells were harvested at 10 days post infection. cccDNA was quantified by qPCR assay and normalized with mitochondria DNA (mtDNA). The relative amounts of cccDNA to that in cells infected with WT HBV from three independent experiments are presented (Mean ± SD) (**C**). HBsAg in culture media was measured by HBsAg-CLIA kit (Autobio) and shown as relative amount in comparison with WT at 4 dpi (Mean ± SD, n = 3) (D). (**E** and **F**) PHHs were infected with WT or Cp mutant HBV at a MOI of 70 GEq. Culture medium was changed every other day. The cells were harvested at 12 days post infection. cccDNA and secreted HBsAg were measured as described above and shown as relative amount in comparison with WT at 2 dpi (Mean ± SD, n = 3). Data were analyzed by two-tailed Student’s t-test (unpaired), ns: no significance; *: *P* < 0.05; **: *P* < 0.01; ***: *P* < 0.001.

It was reported previously by others and us that several type I and type II CpAMs, including GLS4, dose-dependently inhibited cccDNA synthesis during *de novo* HBV infection of hepatocytes [22,23,34]. Mechanistically, it could be due to CpAM-induced structure alteration of nucleocapsids and/or premature uncoating of viral genomic DNA that disrupt the proper transportation of rcDNA into the nuclei for cccDNA synthesis. Whatever the exact mechanism is, it is interesting to know whether the CpAM-resistant Cp also confer resistance to GLS4 inhibition of *de novo* cccDNA synthesis. To achieve this goal, we first prepared HBV virions from the culture media of HepG2 cells transfected with WT or the indicated CpAM-resistant HBV replicons. The yields of WT and mutant HBV were determined by immunoprecipitation of virion particles with antibodies targeting epitopes in preS2 and S region of envelope proteins and quantification of viral DNA by a qPCR assay (S3 Fig) [35]. To determine the effects of the Cp mutations on virion production, the viral particle-associated HBV DNA were normalized to the amounts of intracellular core DNA of the cells transfected with the respective HBV replicons. Comparing to WT HBV, except for Cp T33G mutation that support increased virion production, all the other Cp mutations, particularly T33Q and I105W, significantly reduced the yields of virions (Fig 2A). This result indicates that the amino acid residues at Cp dimer interface not only modulate capsid assembly and pgRNA encapsidation, but also interfere with virion morphogenesis and/or egress.

To determine the infectivity of virions, C3A^NTCP^ or primary human hepatocytes (PHHs) were infected with equal amounts of WT or five Cp-mutant viruses with sufficient yields for the infection assays. HBsAg in the culture media and cccDNA in the infected cultures were determined by ELISA and qPCR assay, respectively. While cccDNA, the first viral product synthesized upon infection, was detectable by qPCR assay only in the cells infected with WT or Cp P25A, P25G or I105F mutant HBV (Fig 2C and 2E), the amounts of cccDNA in the cells infected with Cp P25A, P25G or I105F HBV were apparently less than that in WT HBV-infected cells, as revealed by Southern blot hybridization (S4 Fig). Moreover, comparing to WT HBV-infected cells, the cells infected with Cp P25A HBV secreted slightly reduced amounts of HBsAg, whereas the amounts of HBsAg secreted by cells infected with Cp P25G or I105F HBV are significantly reduced (Fig 2D and 2F). These results indicate that comparing to WT HBV, the infectivity of Cp mutant HBV is similar (P25A), reduced (P25G and I105F) or barely detectable (T33G and T33N). To investigate the mechanism of the reduced infectivity of Cp mutant HBV, secreted virion DNA were extracted and analyzed by Southern blot hybridization (S5 Fig). As anticipated, WT virions contain predominantly mature forms of HBV DNA species, *i*.*e*., rcDNA. Interestingly, while the two Cp mutant HBV (P25A and I105F) with relative higher infectivity contain relatively higher levels of rcDNA, the remaining three Cp mutant virions contain significantly reduced or undetectable amounts of rcDNA. Surprisingly, Cp T33G or I105F nucleocapsids with single-stranded HBV DNA can apparently be enveloped and secreted as virions. These results reinforce the notion that interaction mediated by the selected amino acid residues at Cp dimer-dimer interfaces not only modulate capsid assembly and pgRNA encapsidation, but also profoundly affect the selective envelopment of nucleocapsids for the virion production and strongly impact the infectivity of progeny virions.

**Fig 3:**
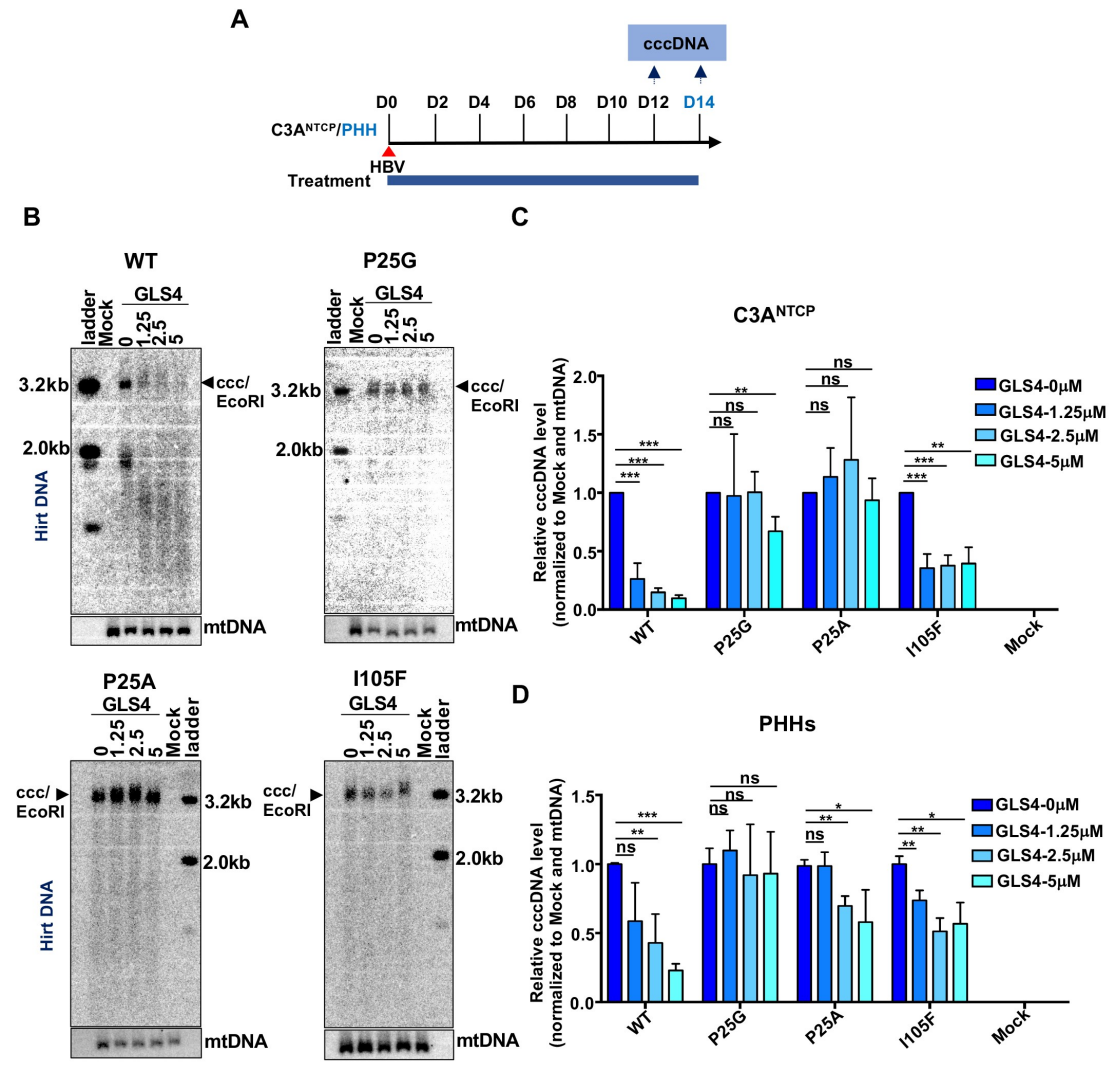
Substitution of Cp residues P25, T33 or I105 confers resistant to GLS4 inhibition of *de novo* cccDNA synthesis. **(A)** Schematic presentation of HBV infection experimental schedule. **(B** and **C)** C3A^NTCP^ cells were infected with the indicated HBV at a MOI of 500 GEq. The cells were mock (DMSO)-treated or treated with the indicated concentrations of GLS4, starting at the time of infection. The culture media were changed every other day. The cells were harvested at 12 days post infection. For detection of cccDNA by Southern blot hybridization, Hirt DNA were denatured at 88°C for 8 min to denature DP-rcDNA into single-stranded DNA and followed by restriction with E*coR*I to convert cccDNA into unit-length double stranded linear DNA, indicated as cccDNA/E*coR*I (B). Quantification of cccDNA was performed with real time PCR assay (C). (**D**) PHHs were infected with the indicated HBV at a MOI of 200 GEq. The cells were mock (DMSO)-treated or treated with the indicated concentrations of GLS4, starting at the time of infection and harvested at 14 days post infection. cccDNA was detected by qPCR assays as described above. Data (Mean ± SD) from three independent experiments were analyzed by two-tailed Student’s t-test (unpaired), ns: no significance; *: *P* < 0.05; **: *P* < 0.01; ***: *P* < 0.001.

Because only three Cp mutant HBV are infectious and produce detectable amounts of cccDNA, we next investigated the effects of GLS4 on cccDNA synthesis in the cells infected with WT and the three Cp mutant HBV. As shown in Fig 3, GLS4 treatment significantly reduced the amounts of cccDNA in a concentration-dependent manner in WT HBV-infected C3A^NTCP^ cells (Fig 3B and 3C) or PHHs (Fig 3D), but did not (P25A or P25G) or less efficiently (I105F) reduced the amount of cccDNA in the cells infected with the three mutant HBV, as revealed by both Southern blot hybridization and quantitative PCR assays.

### CpAM-resistant mutations compromise the biogenesis of HBeAg

**Fig 4:**
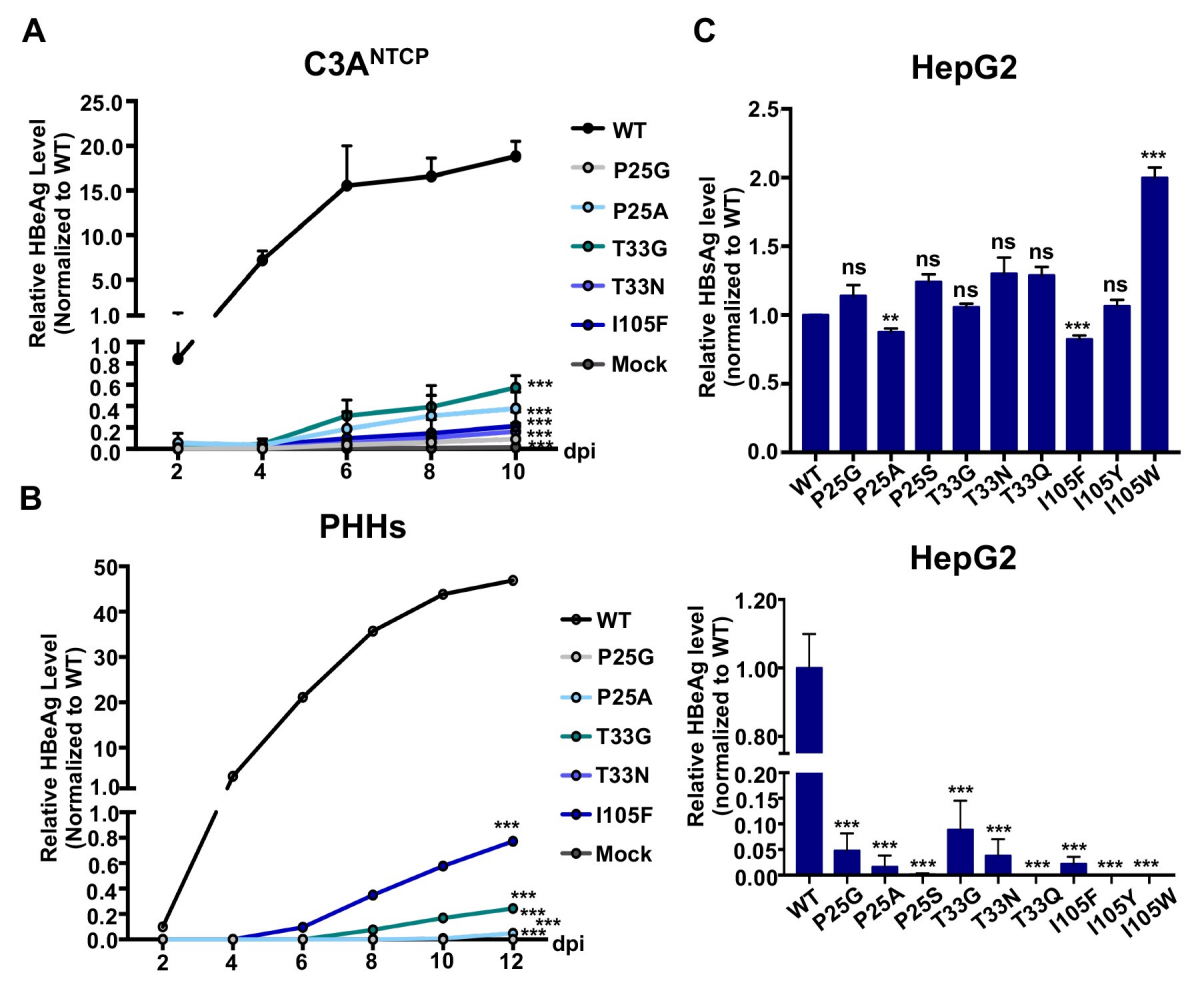
Substitution of Cp residues P25, T33 or I105 reduces HBeAg production. (**A** and **B**) HBeAg in the culture media harvested from the experiments presented in Fig 2D (**A**) and 2F (**B**) measured by HBeAg-CLIA kit (Autobio) and shown as relative amount in comparison with WT at 2 dpi. Data (Mean ± SD) from three independent experiments are presented. (**C**) HepG2 cells were transfected with pHBV1.3 or derived plasmid encoding Cp with the indicated single amino acid substitution and culture media were harvested at 48 h post transfection. HBsAg (upper panel) and HBeAg (lower panel) were measured by CLIA kits (Autobio). The levels of viral antigens were normalized to that in the culture medium of cells transfected with pHBV1.3. Data (Mean ± SD) from three independent experiments were analyzed by two-tailed Student’s t-test (unpaired), ns: no significance; **: *P* < 0.01; ***: *P* < 0.001.

In addition to HBsAg, secretion of HBeAg is another viral biomarker indicating the successful HBV infection of hepatocytes [36,37]. However, to our surprise, while WT HBV-infected C3A^NTCP^ or PHHs secreted significant amounts of HBeAg in a time-dependent fashion, the amounts of HBeAg secreted from the cells infected by the CpAM-resistant HBV were at least 66 folds less than that of WT HBV-infected cells (Fig 4A and 4B). Particularly, although HBsAg produced from Cp P25A HBV-infected cells was only slightly lower than that from WT HBV-infected cells (Fig 2), the hepatocytes infected by this mutant virus produced barely detectable HBeAg (Fig 4A and 4B). To confirm that the CpAM-resistant mutations did compromise HBeAg production, we also measured HBsAg and HBeAg in the culture media of HepG2 cells transfected with WT or the indicated Cp mutant pHBV1.3 replicon plasmid. While all the CpAM-resistant replicons and WT HBV replicon supported similar levels of HBsAg secretion (Fig 4C, upper panel), the amounts of HBeAg secreted from the cells transfected with CpAM-resistant replicons were at least 20 folds less than that from the cells transfected with WT HBV replicon (Fig 4C, lower panel). Because HBeAg is a soluble viral protein derived from pre-C that shares the entire Cp polypeptide at its C-terminal region (S1 Fig), it is, therefore, possible that the CpAM-resistant mutations alter the structure, proteolytic processing and/or trafficking of pre-C and derived proteins, including HBeAg. In agreement with the critical role of these CpAM-resistant amino acid residues in HBeAg biogenesis in the context of pre-C, it was reported that CpAM treatment also inhibited the production of HBeAg [23,29].

### p22, the precursor of HBeAg, exits in both unphosphorylated and phosphorylated forms

**Fig 5:**
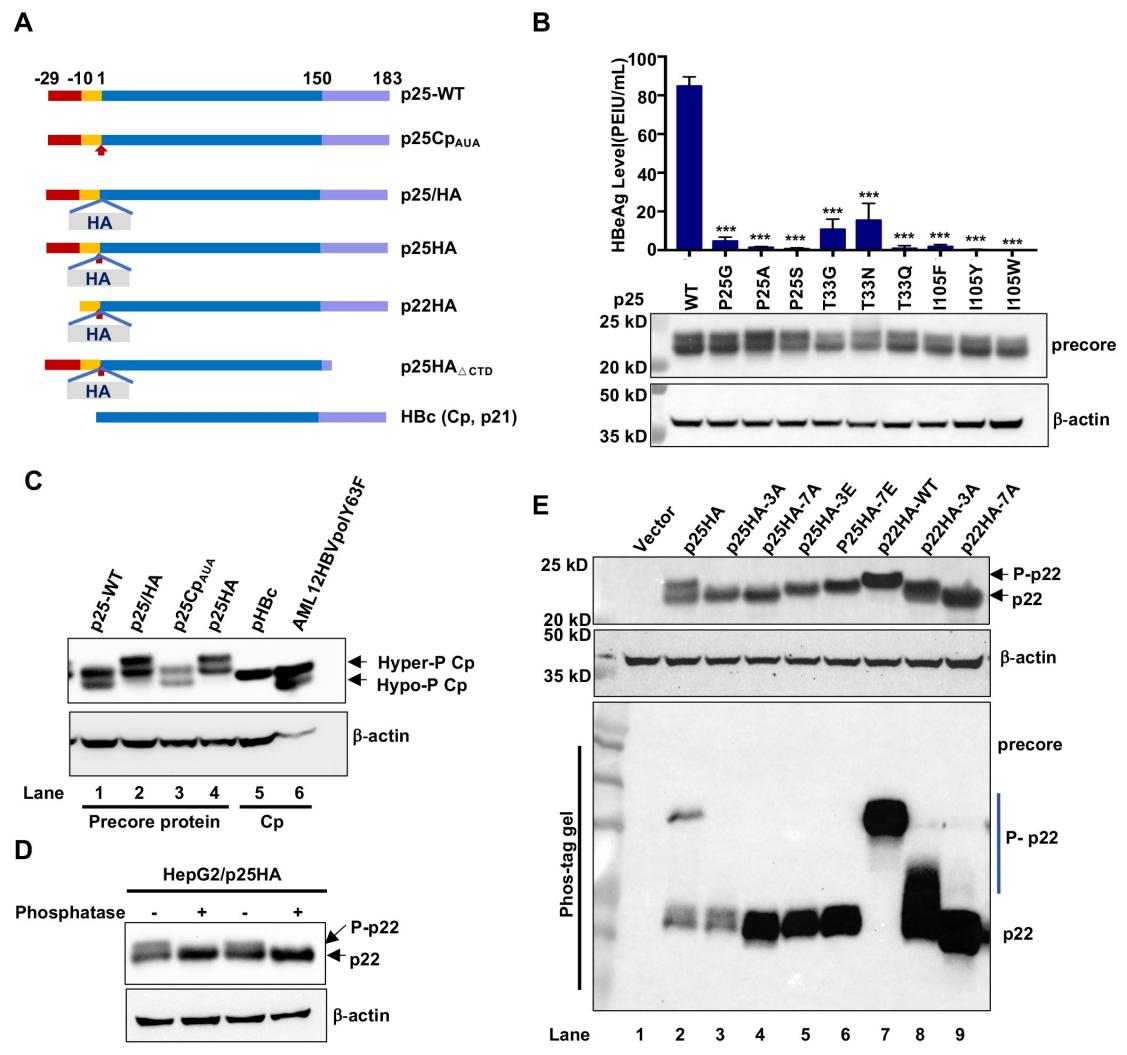
Intracellular precore protein p22 exits as unphosphorylated and C-terminal arginine-rich domain phosphorylated forms. **(A)** Illustration of wild-type precore protein (p25-WT) and mutant/HA-tagged p25 as well as its proteolytically processed products p22 and p17 that are expressed by pXF3H-derived plasmids (see Materials and Methods for detailed description). Red arrow indicates the mutation of Cp starting codon (AUG) to AUA. The gray box indicates the insertion of HA tag at the immediate upstream of Cp starting codon. **(B)** HepG2 cells were transfected with pXF3H-p25-WT and derived plasmid expressing p25 with the indicated single amino acid substitution. The cells were harvested at 48 h post transfection. Intracellular p25-derived proteins were detected by Western blot assay with an antibody recognizing the last 14 amino acid residues at the C-terminus of precore protein and Cp (anti-HBc-170A). HBeAg in culture media was detected by CLIA and results (mean ± SD) from three independent experiments were analyzed by two-tailed Student’s t-test (unpaired), ***: *P* < 0.001. **(C)** HepG2 cells were transfected with a pXF3H-derived plasmid expressing the indicated p25 proteins or pCMV-HBc expressing Cp. Intracellular p25-derived proteins or Cp were detected by Western blot assay with an anti-HBc-170A as the primary antibody. The lysate from AML12HBVpolY63F cells was analyzed in parallel to serve as the control of hyper- and hypo-phosphorylated Cp. **(D)** HepG2 cells were transfected with pXF3H-p25HA, harvested at 48 h post transfection and lysed with core DNA lysis buffer. Aliquots of the cell lysate were mock-treated or treated with Lambda phosphatase at 30°C for 40 min. HBV p22 in the reactions were detected by Western blot assay with anti-HBc-170A. (**E**) HepG2 cells were transfected with a plasmid expressing the indicated precore protein and harvested at 48 h post transfection. The cells were lysed by 1× LDS lysis buffer (contained 2.5% B-ME) and the lysates were resolved by electrophoresis in NuPAGE 12% Bis-Tris Protein Gel (upper panel) and 12% phos-tag gel (lower panel). After blotting onto membranes, p22 were detected by anti-HBc-170A as primary antibody. β-actin served as a loading control.

In order to investigate the mechanism of HBeAg biogenesis and modulation by CpAM-resistant mutations, we first examined intracellular pre-C-derived proteins in HepG2 cells transfected with a plasmid expressing WT (p25-WT) or the indicated mutant pre-C (illustrated in Fig 5A). Western blot analysis with an antibody recognizing C-terminal 14 amino acid residues of Cp revealed two protein species with molecular masses of approximately 22 and 24 kD, respectively, in the cells transfected with the plasmid expressing either WT or mutant pre-C (Fig 5B, lower panel). However, the levels of HBeAg secreted from the cells transfected with plasmids expressing mutant pre-C were 3 to 666 folds less than that from the cells expressing WT pre-C (Fig 5B, upper panel). While these results further confirmed the critical role of residues P25, T33 and I105 in HBeAg biogenesis in the context of pre-C protein expression, the nature of the two intracellular pre-C-derived protein species remained to be determined.

Because the signal peptide of pre-C is co-translationally cleaved by signal peptidase in the ER, the full-length pre-C (p25) is rarely detectable in cells [15]. To facilitate the analysis of pre-C protein processing and determine the nature of the two pre-C-derived protein species detected in Fig 5B, a HA-tag was inserted between precore and Cp regions as previously reported [38] (Fig 5A). Meanwhile, to rule out the internal translation initiation at the Cp starting codon, the starting codon of Cp was mutated to encode isoleucine (I) in plasmids expressing both WT (p25Cp_AUA_) and HA-tagged pre-C (p25HA) (Fig 5A). The pre-C-derived proteins and Cp in the lysates of cells transfected with a plasmid expressing the indicated pre-C or Cp were analyzed by Western blot assay. The cell lysate of AML12HBVpolY63F, an immortalized mouse hepatocyte line supporting efficient assembly of both empty capsids and pgRNA-containing nucleocapsids without expression of pre-C protein [39], served as a control. As reported previously, while only hyper-phosphorylated Cp was detected in HepG2 cells transfected with a plasmid expressing Cp (Fig 5C, lane 5), both hyper- and hypo-phosphorylated Cp were detected in AML12HBVpolY63F cells [39,40] (Fig 5C, lane 6). Mutation of Cp starting codon did not alter the pattern of intracellular pre-C-derived proteins (Fig 5C, comparing lanes 1 and 3) and as anticipated, insertion of a HA tag increased the molecular mass of both pre-C-derived protein species by approximately 1 kD (Fig 5C, comparing lanes 2 and 4). Moreover, both protein species in the cells transfected with plasmid expressing WT pre-C migrated slower than hypo-phosphorylated Cp (Fig 5C, comparing lanes 1 and 6). These results indicate that both of the protein species are derived from pre-C, but not Cp. While the faster migrating species is most likely p22, the slower migrating species could be either full-length pre-C (p25) or phosphorylated p22. Conversion of the slower migrating species into faster migrating species with lambda phosphatase treatment indicates that the slower migrating species is phosphorylated p22 and designated as P-p22 (Fig 5D). Because Cp is phosphorylated at the seven serine/threonine residues in its C-terminal domain (illustrated in S1 Fig) [41–43], we speculated that the same residues might also be phosphorylated in p22. Indeed, substitution of three major or all the seven potential phospho-acceptor residues with alanine (A) either partially (3A) or completely (7A) abolished the phosphorylated species, as revealed by both NuPAGE Bis-Tris protein gel (Fig 5E, upper panel) or phos-tag gel electrophoresis (Fig 5E, lower panel). Interestingly, p22 with phosphomimetic substitutions of S/T with E migrated slowly as phosphorylated p22 in NuPAGE Bis-Tris protein gel, but as anticipated, co-migrated with unphosphorylated p22 in phos-tag gel electrophoresis, due to their inability of binding phos-tag.

### Unphosphorylated and phosphorylated p22 localize in distinct subcellular compartments and have distinct functions

Although it was observed that p22 was phosphorylated by metabolic labeling more than 30 years ago [42], we demonstrated herein, for the first time, that p22 exits in cells as both unphosphorylated and phosphorylated forms. Because all the free Cp dimers are hyperphosphorylated in the cytoplasm [39], we thus hypothesized that the phosphorylation status of p22 may relate to its subcellular localization. Specifically, co-translational translocation of nascent pre-C polypeptide into the ER prevents the phosphorylation of p25 by cytosolic protein kinases, but aborted translocation of p22 after the cleavage of signal peptide or retro-translocation of p22 from the ER into the cytosol results in p22 phosphorylation by cytosolic protein kinases. Consequentially, while the unphosphorylated p22 is transported into the Golgi complex and proteolytically processed by furins to produce p17 for the secretion of HBeAg [44], the phosphorylated p22 may interact with viral (such as Cp) and cellular proteins in the cytoplasm and nuclear to modulate viral replication and cellular functions. In support of this hypothesis, direct expression of mature p22, *i*.*e*., deletion of signal peptide from pre-C protein, in HepG2 cells results in 100% of p22 phosphorylation at its C-terminal domain (Fig 5E, lanes 7, 8 and 9). Moreover, immunofluorescence staining and cell fractionation analysis showed that p22 in cells expressing WT pre-C was primarily detected in the cytoplasm and associated with membranes, signal peptide-deficient p22 diffused in the cytoplasm and predominantly localized in the nuclei (S6 Fig). Interestingly, inhibition of p22 cleavage in HepG2 cells transfected with a plasmid expressing WT p25 by a furin inhibitor induced the intracellular accumulation of unphosphorylated p22, but not phosphorylated p22, and significantly reduced HBeAg secretion (S7A Fig). However, direct expression of signal peptide-deficient pre-C, the mature form of p22, in HepG2 cells did not support HBeAg secretion (S7B Fig). These results thus suggest that the unphosphorylated p22 in the membranous secretary organelles, but not the cytosolic or nuclear phosphorylated p22, is the direct precursor of HBeAg.

**Fig 6:**
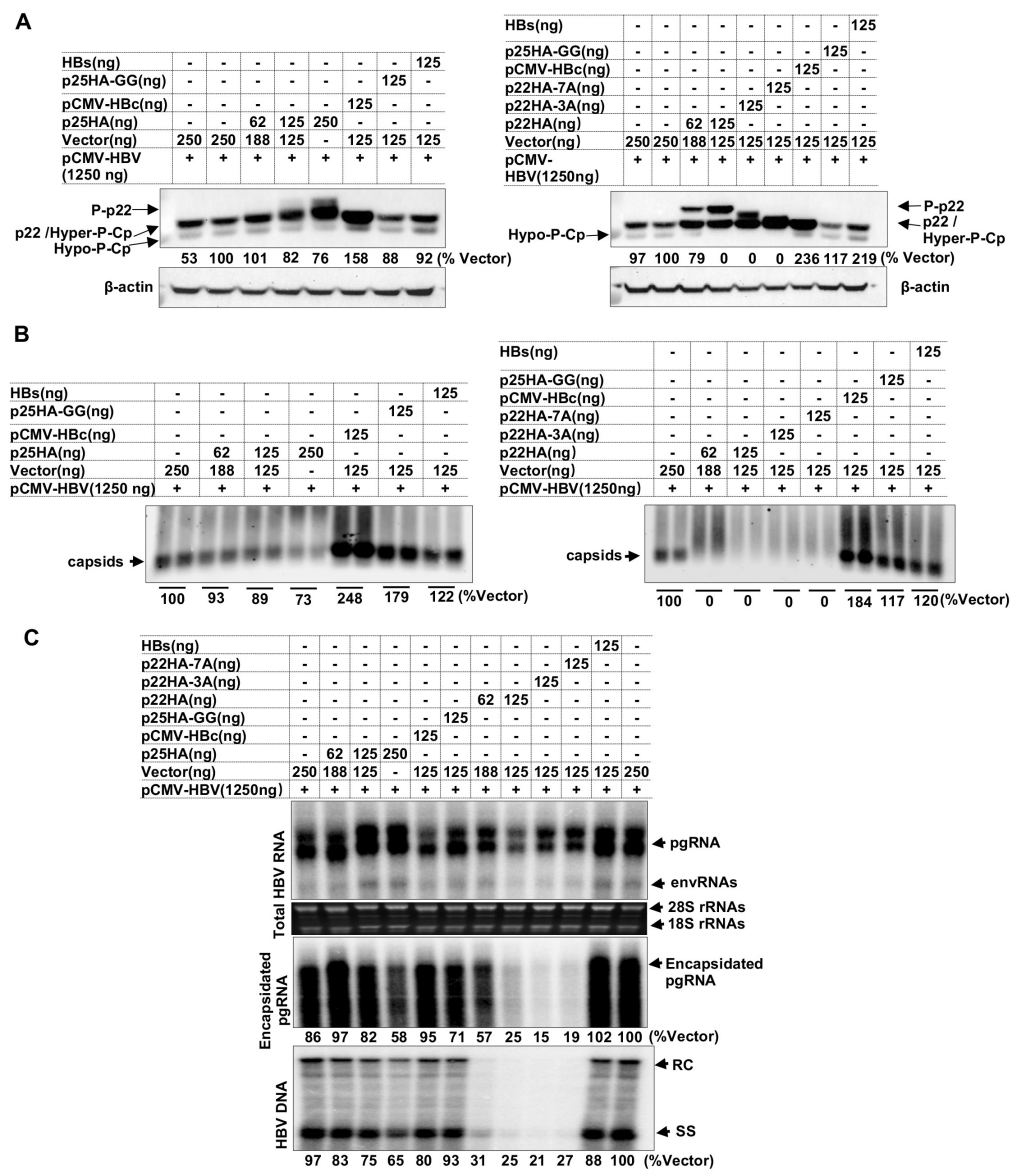
p22 impairs capsid assembly and viral DNA replication in a CTD phosphorylation-independent manner. HepG2 cells cultured in 12-well plates were co-transfected with pCMV-HBV and one of the following plasmids at the indicated amount: pXF3H, pXF3H-p25GG (insertion of GG immediately after pre-C starting codon, which results in the frame shift mutation of p25 to serve as a negative control), pXF3H-HBs (expressing HBsAg), pXF3H-p25HA or pXF3H-p22HA, pXF3H-p22HA-3A, pXF3H-p22HA-7A. The cells were harvested at 72 h post transfection. **(A)** Intracellular HBV precore and core proteins were detected by Western blot assay with anti-HBc-170A as primary antibody. β-actin served as a loading control. **(B)** Intracellular capsids were detected by particle gel assay. **(C)** Total intracellular HBV RNA as well as encapsidated pgRNA were detected by Northern blot hybridization. 28S and 18S rRNA served as loading controls. HBV core DNA were analyzed by Southern blot hybridization. RC, relaxed circular DNA. SS, single stranded DNA. The gray values of hypo-phosphorylated Cp **(A)**, capsids **(B)**, encapsidated pgRNA and HBV DNA **(C)** were quantified by Image J and presented as the percentage of that in cells transfected with control vector plasmid.

In agreement with previous reports [45,46], co-expression of pre-C in HepG2 cells transfected with pCMV-HBV, which only transcribes pgRNA, but not pre-C mRNA, due to cloning strategy [38], dose-dependently inhibited HBV replication, most likely *via* interaction with Cp to disrupt capsid assembly (Fig 6). Specifically, in agreement with our previous findings that the dephosphorylation of Cp occurred during pgRNA encapsidation [39,40], expression of WT pre-C (Fig 6A and 6B, left panel) or signal peptide-deleted pre-C (mature p22) (Fig 6A and 6B, right panel) reduced the levels of dephosphorylated Cp (Fig 6A), capsids (Fig 6B) as well as encapsidated pgRNA and viral DNA replication intermediates in a dose-dependent manner (Fig 6C). Interestingly, p22 suppression of capsid assembly, pgRNA encapsidation and viral DNA replication did not depend on phosphorylation in its C-terminal domain, as demonstrated by the strong inhibition of HBV replication by p22-3A and p22-7A.

In summary, the results presented in this section support the hypothesis that while p22 translocated into the ER lumen are unphosphorylated and proteolytically processed by furins into p17 that is subsequently secreted out of the cells as HBeAg, the cytosolic and nuclear p22 are hyperphosphorylated and interact with Cp to disrupt capsid assembly and viral genome replication.

### Identification of amino acid residues critical for HBeAg biogenesis

**Fig 7:**
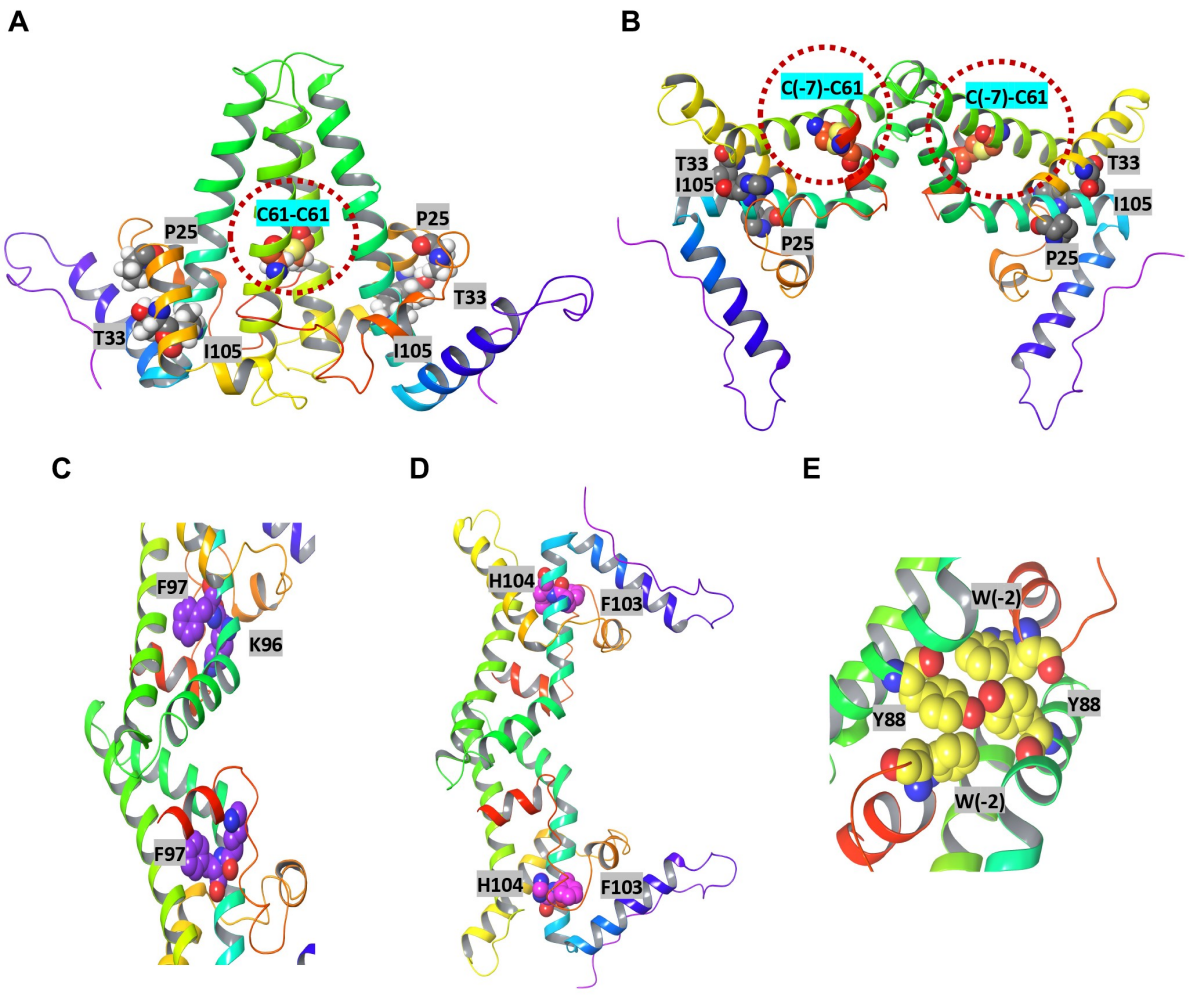
Structure features of Cp and p17. Comparing to Cp dimer (**A**), the largest structural changes of HBeAg (**B**) are found in helix a3 and a4, located at the intradimer interface. Cysteine(-7), located on the amino-terminal extension of HBeAg (3V6Z) (magenta spheres) can form a disulfide with cysteine 61 (yellow spheres). To accommodate this peptide, the monomers are rotated 140° about the intradimer interface from their orientation in Cp dimer. In Cp dimer, the monomers are parallel, and an intradimer Cys61-Cys61 disulfide can form. The W(-4)-W62-K96-F97 pi-pi bond (**C**) and Y6-F9-F103-H104 interaction (**D**) critical for monomer stability as well as the inter-monomer W(-2)-Y88 interaction (**E**) are highlighted.

The results presented above showed that although CpAM-resistant Cp mutations in the context of pre-C protein significantly reduced HBeAg secretion, they did not apparently alter the levels of intracellular unphosphorylated and phosphorylated p22 (Fig 5B). This finding implies that the mutations do not interfere with the cleavage of pre-C signal peptide and stability of p22. Instead, the mutation may impair the production, stability, secretion and/or antigenicity of p17. As shown in S1 Fig, except for the 10 amino acid propeptide at its N-terminal region, HBeAg (p17) shares the entire assembly domain and linker region with Cp. Interestingly, it was reported that p17 monomer fold is essentially the same as that of Cp [17]. However, an intramolecular disulfide bond is uniquely formed between C(-7) in the propeptide and C61 at helix 3 in p17 monomer and was reported to be essential for the secretion of HBeAg [47,48]. As illustrated in Fig 7, while Cp dimerize through the in parallel pairing of two helical hairpins to form a four-helix bundle (Fig 7A), the propeptide loop in p17 monomer makes stabilizing hydrophobic contacts with the central part of the α3-α4 surface of its own polypeptide chain, which sterically blocks the formation of a Cp-like dimer. Instead, p17 dimerize *via* the hydrophobic contacts of nearly antiparallel α3b helices of two monomers (Fig 7B). Unlike Cp dimer, the α4a helices do not interact with each other, but interact with the exterior surface of the partner’s propeptide *via* hydrophobic contacts. Not surprisingly, p17 dimers cannot assemble into capsids under physiological condition, but are secreted as HBeAg [49]. Since residues P25, T33 and I105 are not involve in p17 dimerization, it is difficult to envisage how the CpAM resistant mutations of these residues impair HBeAg biogenesis.

In order to understand the structural basis of HBeAg biogenesis, in addition to these CpAM resistant mutations, single amino acid substitutions were also made in the context of p25HA (Fig 5A) to mutate the residues that are critical for (*i*) the intramolecular disulfide bond formation, C(-7) and C61 (Fig 7B); (*ii*) monomer stabilization through intramolecular pi-pi interactions, W(-4)-W62-K96-F97 (Fig 7C) or Y6-F9-F103-H104 interaction network (Fig 7D) and (*iii*) dimerization through hydrophobic interactions between propeptide and α4 helices of partner subunit, W(-2) and Y88 (Fig 7E). A few residues (P45, P50, V124, I126, P129 and S141) predicted to play no role in p17 dimerization and stability were included in the mutagenesis analysis as negative controls.

**Fig 8:**
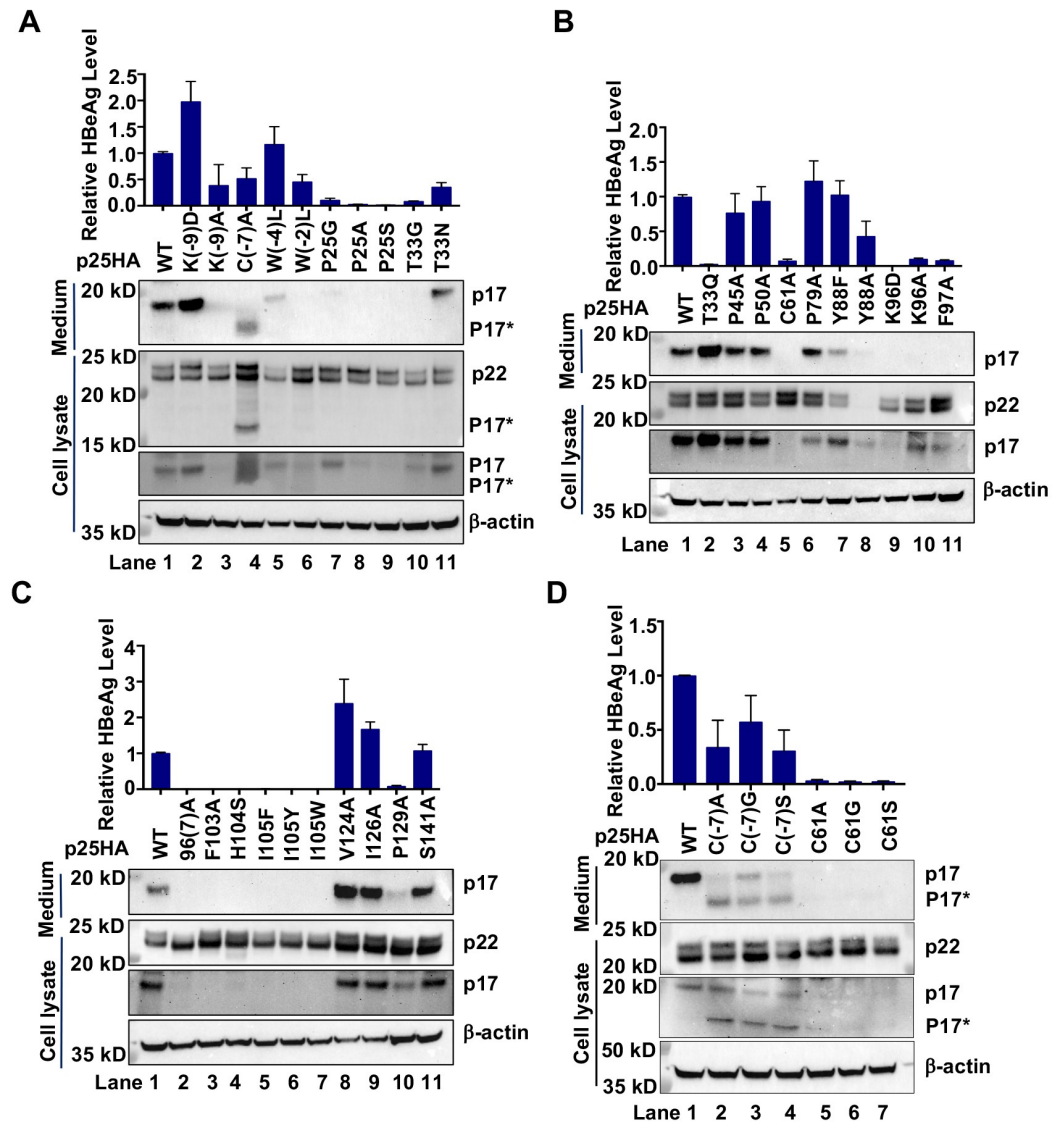
Mutagenesis analysis of HBeAg biogenesis. (**A** to **D)**. HepG2 cells were transfected with pXF3H-p25HA derived plasmid expressing WT or mutant p25HA with the indicated single or double amino acid residue substitution and harvested at 48 h post transfection. HBeAg in culture media were measured by CLIA kit. Secreted p17 was detected by IP-Western blot assay. Intracellular p22 and p17 were detected by Western blot assays with antibody against HA tag. β-actin served as a loading control. The relative levels of HBeAg in culture medium to that of cells expressing WT p25HA from three independent experiments are presented (Mean ± SD).

In agreement with previous reports, substitution of C61 by serine(S), alanine(A) or glycine(G) abolished HBeAg secretion [47,50]. However, substitution of C(-7) by S, A or G only modestly reduced the secretion of HBeAg (Fig 8A, 8B and 8D). Western blot analysis of intracellular and secreted pre-C-derived proteins indicated that while mutation of C(-7) resulted in the production of slightly smaller p17 (p17*), the cells expressing pre-C with C61 substitutions accumulated similar levels of p22, but undetectable levels of intracellular and secreted p17 (Fig 8B and 8D). The findings from the analyses of other single amino acid substitutions (Fig 8A–8C) can be summarized as follows. *First*, except for Y88A mutation that significantly reduced the amount of p22, all the other mutations did not apparently or only modestly (Y88F and K96D) alter the amounts of p22. Reproducible alterations on the relative amounts of unphosphorylated and phosphorylated p22 among all the mutations were not observed, suggesting that these mutations do not impair the ER translocation of pre-C. *Second*, while the levels of HBeAg signals detected by ELISA and secreted p17 detected by Western blot assay were generally consistent for the majority of mutant pre-C proteins, T33Q mutation allowed the efficient secretion of p17, but showed an undetectable level of HBeAg by ELISA. On the contrary, HBeAg signals detected by ELISA for K(-9)A, W(-4)L, W(-2)L and Y88A mutant pre-C protein were relatively higher than the amounts of secreted p17 revealed by Western blot assay. The results indicate that these substitutions abolished or possibly enhanced the recognition (or binding) of secreted p17 by the antibody against HBeAg in the ELISA assay. *Third*, many mutations reduced the levels of intracellular p17. However, the level of secreted p17 was generally consistent with its intracellular level of p17, suggesting that the mutations most likely impair the intracellular production and/or stability of p17, but not its secretion process.

### Substitution of single amino acid residues differentially affects the production and intracellular stability of p17

**Fig 9:**
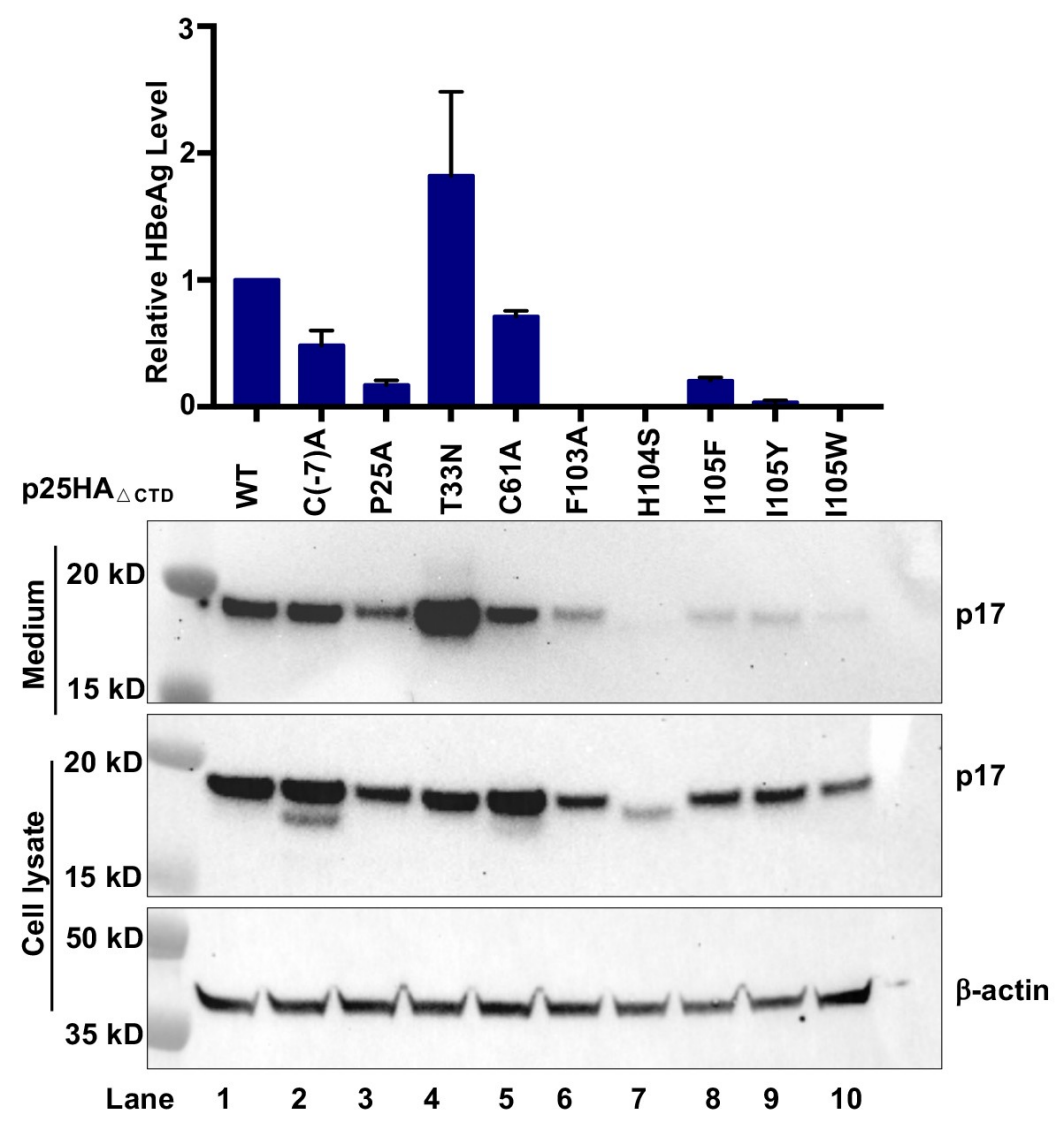
Substitution of selected amino acids in propeptide and the assembly domain of Cp reduced the steady-state levels of intracellular p17. HepG2 cells were transfected with plasmid pXF3H-p25HA_ΔCTD_ or derived plasmid expressing WT or mutant p17HA with the indicated single amino residue substitution and harvested at 48 h post transfection. Intracellular p17 were detected by Western blot assay with an antibody against HA tag. β-actin served as a loading control. The secreted p17 were measured by IP-Western blot assay. HBeAg levels were measured by CLIA kit and the results (mean ± SD) from two independent experiments are presented.

To further investigate the mechanism of selected single amino acid substitutions on the inhibition of HBeAg biogenesis, we intended to distinguish whether a specific mutation impairs p17 production from its precursor p22 or reduces the stability of p17 in hepatocytes. To this end, we expressed a HA-tagged, C-terminally truncated (at residue R154) precore protein (p25HA_ΔCTD_, illustrated in Fig 5A) without or with a desired single amino acid substitution in HepG2 cells. As anticipated, cleavage of signal peptide from this protein in the ER produced the authentic p17 and secreted out of the cells as HBeAg (Fig 9). Interestingly, while C61A, C(-7)A and T33N substitutions did not apparently reduced the levels of intracellular and secreted p17, substitutions of residue P25, F103, H104 or I105 significantly reduced the levels of both intracellular and secreted p17. Because all those single amino acid substitutions did not apparently reduced the levels of intracellular p22 when expressed in the context of full-length precore protein (Fig 8), it is reasonable to conclude that suppression of HBeAg secretion by these single amino acid substitutions is due to either the inhibition of p17 production (such as C61A substitution) or reducing the apparent stability of intracellular p17 (such as P25A and F105 substitutions).

Taking together, the mutagenesis analyses in full-length and C-terminally truncated precore proteins clearly indicate that substitution of selected single amino acid residues can impair p22 stability, p17 biogenesis or stability, or antigenicity of HBeAg (Figs 8 and 9). Particularly, the results generally support the important role of the residues critical for intramolecular disulfide bond formation, p17 monomer and dimer stability in HBeAg biogenesis. However, regarding the mechanism by which the CpAM-resistant mutations reduced HBeAg production, our results indicate that substitution of residues P25 or I105 most likely destabilizes intracellular p17. T33Q substitution apparently abolished the HBe antigenicity of p17 and could not be recognized by anti-HBe in ELISA assay, whereas T33N and T33G mutation most likely altered p17 biogenesis and stability, respectively.

### A significant portion of intracellular and secreted p17 does not have an intramolecular disulfide bond

As pointed out above that although it was reported that the proper folding and dimerization of p17 is critical for its secretion, the phenotypes of the many mutations in the various regions of p17 cannot be interpreted with the HBeAg structure model. These findings promoted us to investigate the folding and oligomerization status of p17. A comparative analysis of intracellular and secreted p17 from HepG2 cells transfected with a plasmid expressing C-terminally truncated WT or C(-7)A precore protein by SDS-PAGE in non-reducing and reducing condition clearly indicated the existence of two forms of p17 monomers under the non-reducing condition in cells expressing WT p17 (S8 Fig). Conversion of the faster migrating species into the slower migrating species under the reducing condition as well as the lack of faster migrating species in the cells expressing C(-7)A p17 indicates that the faster migrating protein was p17 monomer with intramolecular disulfide bond between C(-7) and C61. The results thus revealed that the intramolecular disulfide bond was only formed in a portion of the intracellular and secreted p17 monomers and approximately 37% of intracellular p17 did not have intramolecular disulfide bond (S8 Fig). This finding suggests that under certain conditions, the reduced p17 monomers may form dimers structurally similar to Cp dimers (Fig 7A). It had been shown that like Cp dimers, the reduced p17 dimers are competent to assemble into capsids in E. coli and in *in vitro* assembly system [51,52].

### GLS4 inhibits HBeAg production by reducing intracellular p17, but not p22

**Fig 10:**
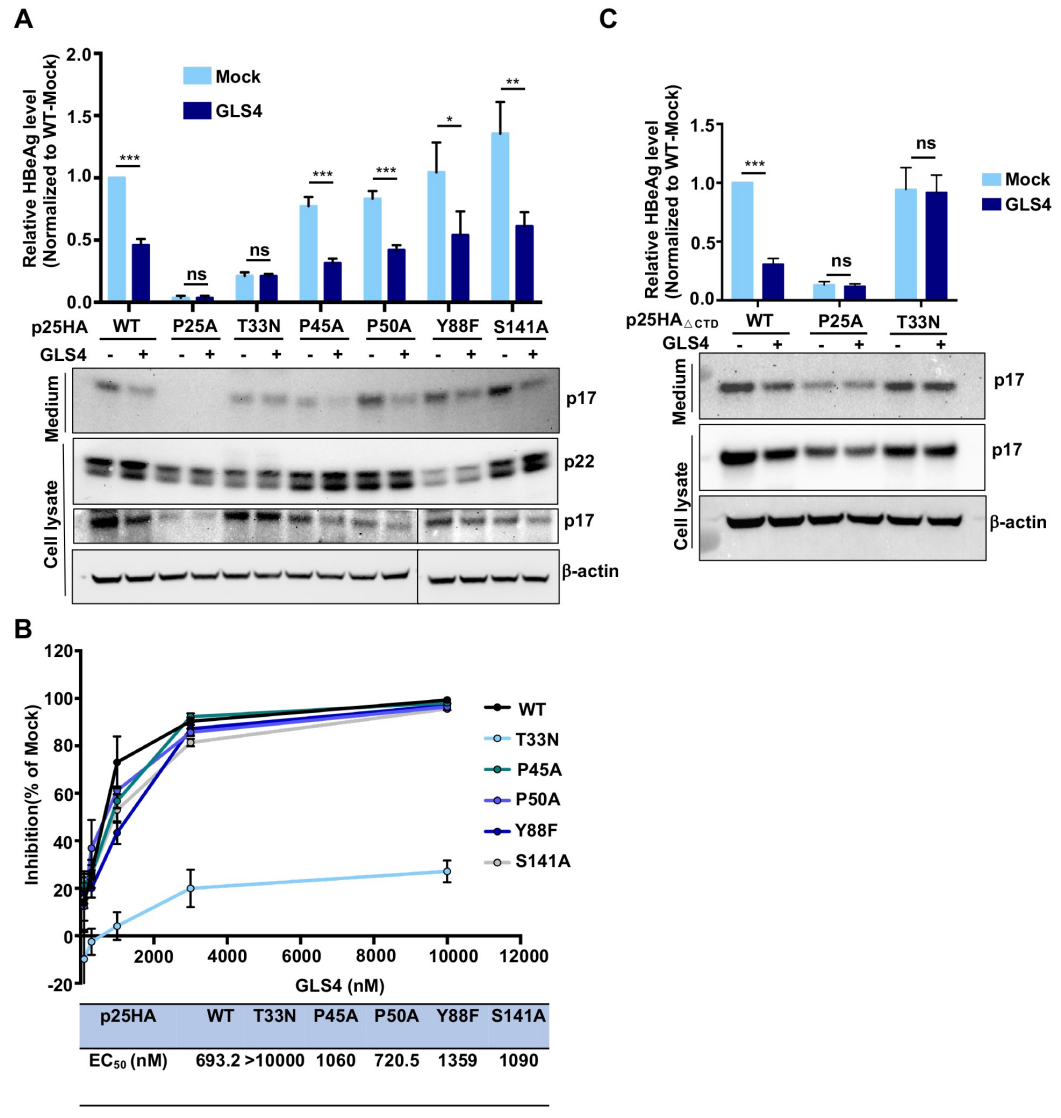
P25A and T33N mutations in precore polypeptide confer resistance to GLS4 inhibition of HBeAg secretion. **(A)** HepG2 cells were transfected with pXF3H-p25HA and derived plasmid expressing p25HA with the indicated single amino acid residue substitution and mock (DMSO)-treated or treated with GLS4 (1 μM), starting at 6 h post transfection, for 48 h. Intracellular precore-related proteins, p22 and p17, were detected by Western blot assay with an antibody against HA tag. β-actin served as a loading control. The secreted p17 were detected by IP-Western blot assay. HBeAg in the culture medium was measured by CLIA kit. **(B)** HepG2 cells transfected with pXF3H-p25HA and derived plasmid expressing p25HA with the indicated amino acid substitution were mock-treated or treated with three folds serial dilution of GLS4, starting at 6 h post transfection, for 48 h. HBeAg in the culture medium was measured by CLIA kit. Result for Western blot was shown as one representative image. HBeAg levels (mean ± SD) from three independent experiments were analyzed by two-tailed Student’s t-test (unpaired). ns: no significance; *: *P* < 0.05; **: *P* < 0.01; ***: *P* < 0.001. The dose-response curve and the EC_50_ for inhibition of HBeAg were calculated by graph prism 7.0. **(C)** HepG2 cells were transfected with pXF3H-p25HA_ΔCTD_ and derived plasmid with P25A or T33N mutation. The cells were mock-treated or treated with GLS4 (1 μM), starting at 6 h post transfection, for 48 h. Intracellular p17 was detected by Western blot assay with an antibody against HA tag. β-actin served as a loading control. The secreted p17 were detected by IP-Western blot assay. HBeAg in the culture medium was measured by CLIA kit.

The existence of reduced p17 monomer in hepatocytes and the capability of reduced p17 dimers for assembly of capsid-like structures evoke a possibility that the reduced p17 monomers may form homodimers in the Golgi complex and secretory vesicles of hepatocytes. Although p17 does not normally assemble into capsids in hepatocytes, we speculate that CpAMs may induce the reduced p17 dimers to assemble into non-capsid polymers in hepatocytes by binding to the “HAP” pocket between p17 dimer-dimer interface during the assembly process, which consequentially reduces p17 secretion as HBeAg. In support of this hypothesis, it was demonstrated that a HAP compound can misdirect the assembly of reduced p17 dimers into non-capsid aggregates *in vitro* and a CpAM-resistant Cp mutation also conferred resistance to HAP inhibition of HBeAg secretion [29]. To investigate whether GLS4 inhibits HBeAg biogenesis by directly targeting intracellular p17, the effects of GLS4 on intracellular precore-derived proteins and secreted p17/HBeAg were evaluated in HepG2 cells expressing WT and indicated mutant pre-C proteins (p25HA). Interestingly, GLS4 treatment did not alter the levels of p22 in cells expressing either WT or all the mutant pre-C proteins, but reduced the levels of intracellular and secreted p17 in cells expressing WT and mutant precore proteins, except for precore proteins with CpAM-resistant mutations P25A and T33N (Fig 10A). A strong resistance of T33N mutation on GLS4 inhibition of HBeAg production was further validated by a dose-response assay (Fig 10B). These results indicate that GLS4 inhibits HBeAg production by targeting intracellular p17, but not p22. In support of this notion, GLS4 treatment of HepG2 cells expressing C-terminally truncated WT or mutant precore protein p25HA_ΔCTD_ with P25A or T33N mutation significantly reduced the levels of intracellular and secreted WT p17, but not CpAM-resistant mutant p17 (Fig 10C). In agreement with the modest reduction of p17 in GLS4 treated cells, we further demonstrated that GLS4 treatment slightly accelerated p17 decay in HepG2 cells (S9 Fig).

## Discussion

Assembly of HBV capsids is driven by hydrophobic interaction of Cp dimers. Interestingly, various structurally distinct CpAMs misdirect the assembly of Cp dimers into either morphologically normal empty capsids or aberrant non-capsid Cp polymers by binding to the hydrophobic HAP pocket between Cp dimer-dimer interface [8,53,54]. Crystallography and Cryo-EM structure analyses indicate that different CpAMs bind different sub-pockets and interact with common as well as distinct residues at the HAP pocket [20,21,55,56]. Apparently, it is the unique binding pose and specific interaction with distinct residues at the HAP pocket that determine the alterations of a CpAM on Cp dimer assembly kinetics and morphology/structure of assembled products [57]. Consistent with the critical role of Cp dimer-dimer interaction in capsid assembly and pgRNA-Pol encapsidation, mutagenesis analyses clearly indicate that disruption of Cp dimer interaction by mutation of selected residues at the HAP pocket impairs capsid assembly and/or pgRNA packaging in a fashion similar to that of CpAM-misdirected Cp dimer assembly [26,58,59]. Not surprisingly, many of the CpAM-resistant Cp mutations severely compromised pgRNA encapsidation and viral DNA synthesis [32,54,60] (Fig 1). Moreover, while single amino acid mutations at the HAP pocket usually confer resistance to selected chemotypes of CpAMs [20,32,60,61], our systematic mutagenesis study of 25 amino acid residues at the wall of HAP pocket identified that several mutations of residue P25, T33 or I105 conferred strong resistance to the inhibition of HBV DNA replication by heteroaryldihydropyrimidines (HAP) as well as four chemotypes of type II CpAMs [26]. Importantly, seven out of the nine mutations supported relatively high levels of pgRNA packaging and DNA synthesis, which allowed investigating the role and mechanism of these residues in other steps of viral replication. Indeed, we demonstrated in this study that mutations of these residues impair not only the pgRNA packaging (S2 Fig) and DNA replication (Fig 1A), but also the stability (Fig 1D) and envelopment (Figs 2 and S3 and S5) of nucleocapsids, virion infectivity and cccDNA biosynthesis (Figs 2 and S4).

Although prior studies demonstrated that residue I126 at the vicinity of HAP pocket play a critical role in virion production [12,13], our results further showed that the selected mutations of residues P25, T33 or I105 reduced the yields of complete virions (Fig 2A). Intriguingly, although Southern blot analysis indicated that rcDNA was synthesized in the cells supporting all the Cp mutant HBV examined (Fig 1A), P25G and T33N mutations excluded the envelopment of rcDNA-containing nucleocapsids. On the contrary, T33G and I105F mutations enabled the envelopment of immature nucleocapsids with single-stranded DNA (S5 Fig). It was reported recently that the conformation of a hydrophobic pocket at the bottom of spikes on the surface of capsids may be critical for specific interaction with envelop proteins and virion morphogenesis [62]. It is thus possible that these Cp mutations altered the conformation of this pocket and consequentially reduced the efficiency and specificity of nucleocapsid envelopment. The reduced infectivity of the Cp mutant HBV is generally consistent with their reduced capability to synthesize cccDNA (Figs 2 and S4), which could be due to either the impaired uncoating of DNA contents or the reduced efficiency to convert the immature forms of genomic DNA into cccDNA in infected hepatocytes.

While the antiviral activity and mechanism of CpAMs on capsid assembly have been extensively investigated, the mode of CpAM actions on other steps of viral replication, particularly cccDNA synthesis, remains to be thoroughly determined [8]. Although it was well documented that HAPs can bind to the HAP pockets of *in vitro* assembled capsids and induce capsid structure alteration or disassembly [63,64], treatment of capsids purified from HBV-replicating hepatoma cells with HAP compounds only induce the electrophoresis mobility shift [24], an indication of global structure alteration of capsids [32]. Intriguingly, we showed that CpAM treatment of capsids purified from HBV-replicating cells preferentially induces the uncoating of nucleocapsids with double-stranded DNA [22,30]. A recent report also revealed that ABI-H0731 treatment promoted the uncoating of virion DNA in the infected hepatocytes [61]. Taken the advantage of CpAM-resistant Cp mutations supporting HBV replication and virion production, we showed in this study that the Cp mutations conferring resistance to GLS4 suppression of capsid assembly and DNA synthesis also conferred resistance to its induction of capsid structure alteration and mature nucleocapsid disassembly as well as its suppression of cccDNA synthesis (Figs 1–3). We have thus obtained genetic evidence supports that GLS4 (and presumably other CpAMs) can bind to the HAP pocket and interact with the same key Cp residues for misdirection of capsid assembly to alter the structure of capsids/nucleocapsids and induce the disassembly of mature nucleocapsid in hepatocytes. Considering that I105F mutation conferred resistance to GLS4-induced mature nucleocapsid disassembly (Fig 1D), but not to GLS4-induced capsid mobility shift (Fig 1C), it is possible that the lesser resistance of Cp I105F mutant HBV to GLS4 inhibition of cccDNA synthesis implies that GLS4-induced relatively subtle capsid structure alteration contributes, at least in part, to its inhibition of cccDNA synthesis.

The most striking finding of this study is that the CpAM resistant mutations compromise HBeAg biogenesis. Mechanistically, the CpAM-resistant mutations in the context of precore protein did not interfere with p22 biogenesis, but reduced the accumulation of intracellular p17 or altered the epitope recognized by the antibody against HBeAg in ELISA. Importantly, we demonstrated for the first time that intracellular p22 existed as both unphosphorylated and phosphorylated forms (Fig 5). While the unphosphorylated p22 localizes in the membranous secretary organelles and is the precursor of HBeAg, p22 in the cytosol and nuclei is hyperphosphorylated at the C-terminal domain and interacts with Cp to disrupt capsid assembly and viral DNA replication (Figs 6 and S6 and S7). Although it has been taken for granted for several decades that HBeAg biogenesis follows the well-characterized protein secretion pathway, the results presented herein indicate that the process can be disrupted by many single amino acid substitutions. On many occasions, the mutagenesis phenotypes cannot be interpreted by the known structure model of HBeAg. Specifically, although it is well established that the intramolecular disulfide bond between C(-7) and C61 is essential for the secretion of HBeAg [47,50], our results clearly demonstrated that substitution of C(-7) and C61 with residues A, S or G, respectively, differentially impacts intracellular p17 production (Fig 8D). Moreover, while the mutations disrupting the intramolecular pi-pi interactions of p17 monomer significantly reduced the accumulation of intracellular p17 and HBeAg secretion, it remains to be known how the mutations of residues P25 and I105 reduced the accumulation of intracellular p17. Although the reduced p17 intracellular accumulation in cells expressing C-terminally truncated pre-C (p25HA_ΔCTD_) strongly suggests that these mutations may destabilize p17, treatment of the cells with proteosome and/or autophagy-lysosome inhibitors only modestly rescued the levels of selected, but not all these mutant p17 (S10 Fig). These findings thus raise more questions on the biochemical mechanism of HBeAg biogenesis and its regulation by viral and host cellular factors.

In principle, in addition to precore stop codon and basal core promoter mutations [65], the loss of HBeAg or even HBeAg to anti-HBe seroconversion is possible to be achieved by immune selection of HBV with specific Cp mutations that support HBV replication, but are unable to secret HBeAg. In fact, sequence alignment analysis of residues P25, T33 and I105 from 16,432 HBV core protein sequences deposited in GenBank with the method described previously [32] indicates that four of the CpAM-resistant Cp mutations reported herein, P25S, T33N, I105F and I105W, exist at a frequency of 0.019, 0.057, 0.032 and 0.019, respectively. It is thus possible that patients bearing HBV with these Cp substitutions may have undetectable or reduced levels of HBeAg. However, because the majority of these Cp substitutions significantly reduced the viral replication fitness (Figs 1 and 2), HBV variants with those mutations may not be the dominant quasi-species and therefore do not significantly affect the levels of HBeAg. Interestingly, we found that mutation of several other Cp residues significantly compromised HBeAg biogenesis (Fig 8). A recent clinical study also revealed that Cp W62R substitution significantly reduced HBeAg production [66]. Therefore, further clinical studies are warranted to determine the role of Cp mutations in the level of HBeAg as well as HBeAg to anti-HBe seroconversion. Considering the proposed immune regulatory roles of HBeAg [65], these Cp mutations might have impacts on HBV pathogenesis.

It is rather interesting that many chemotypes of CpAMs inhibited HBeAg production in cultured hepatocytes [23] or in HBV hydrodynamic mice model in vivo [29]. Based on the observation that treatment of HBV-replicating hepatocytes with HAP_R01 induced the aggregation of p22 and Cp for subsequent degradation in the cytoplasm or accumulation in the nuclei of hepatocytes, it was concluded that the CpAM suppression of HBeAg secretion by depletion of its precursor p22 through induction of its co-assembly with Cp [29]. However, while this explanation is consistent with the speculated HAP pocket binding at the P22/Cp dimer-dimer interface and shared resistance profile with modulation of capsid assembly, it is inconsistent with the results that CpAMs efficiently inhibited HBeAg production in cells expressing pre-C protein alone (Figs 10A and 10B and S9) [23]. Moreover, Cp and p22 only co-exist in the cytosol and nuclei, but not in the ER and Golgi complex. We demonstrated in this study that the cytosolic and nuclear p22 is not the precursor of HBeAg (S7 Fig). Therefore, it is unlikely that the observed co-assembly of precore protein and Cp is responsible for the inhibition of HBeAg production by CpAMs. In fact, the results presented in Fig 10C clearly demonstrated that when expressed as p17 alone in the ER and Golgi complex in HepG2 cells, GLS4 treatment significantly reduced the intracellular p17 and reduced HBeAg production. However, both P25A and T33N mutant p17 were resistant to GLS4 treatment. Our results thus clearly mapped the target for CpAM inhibition of HBeAg production at p17 in the secretary pathway. Because not all the p17 in hepatocytes have intramolecular disulfide bond between C(-7) and C61 (S8 Fig), we thus speculated that similar to its action on Cp dimers, GLS4 accelerated the assembly of reduced p17 dimers into non-capsid polymers and HBeAg secretion was subsequently reduced.

In conclusion, we demonstrated in this study that through mediation of Cp dimer interaction in capsid assembly, pgRNA packaging, nucleocapsid maturation and disassembly, the amino acid residues at the wall of the hydrophobic HAP pocket at Cp dimer-dimer interface play critical roles in all these steps of HBV replication. Interestingly, these residues also modulate HBeAg biogenesis *via* maintaining the stability of p17 by unknown mechanisms. Similarly, through modulating the interaction of Cp dimers as well as reduced p17 dimers by binding to these amino acid residues at HAP pocket, CpAMs impair multiple steps of HBV replication involving Cp and inhibit HBeAg biogenesis. Further investigation on the mechanism of CpAM interaction with Cp in the context of nucleocapsids and reduced p17 dimer by molecular binding and biophysical analyses should provide much needed molecular insights on the mode of CpAM action and pave the way toward the discovery of novel Cp/p17-targeting antiviral agents that can more efficiently inhibit cccDNA synthesis and induce host antiviral immune responses to achieve the cure of CHB [6].

## Materials and methods

### Cell culture

Human hepatoblastoma cell line HepG2 was purchased from ATCC (HB-8065). C3A (ATCC CRL-10741) is a clonal derivative of HepG2 that was selected for strong contact inhibition of growth and high albumin production [67]. C3A^NTCP^ is a C3A-derived cell line stably expressing human NTCP [68]. HepG2 and C3A^NTCP^ were cultured in Dulbecco’s Modification of Eagle’s Medium (DMEM) (Corning) supplemented with 10% fetal bovine serum (FBS) (Gibco), 100 U/mL of penicillin, 100 μg/mL of streptomycin. Primary human hepatocytes (PHHs) were purchased from Yecuris, Tualatin, OR, USA and cultured with HCM medium (Lonza, MD, USA, Cat. No. CC-4182).

### Chemicals and antibodies

Proteasome inhibitor MG-132 (Cat. No. 474790), autophagy inhibitor chloroquine (CQ) (Cat. No. C6628), the protein synthesis inhibitor cycloheximide (CHX) (Cat. No. C7698), furin I inhibitor (Cat. No. 344930) were purchased from Sigma. GLS4 was provided by Arbutus Biopharma, Inc. Information for antibodies used in this study is shown in S4 Table.

### Plasmids

Plasmid pCMV-HBV expressing HBV pgRNA under the control of CMV immediate early (IE) promoter and HBV replicon pHBV1.3 as well as pCMV-HBc expressing full length HBV Cp were reported previously [69–71]. Plasmids pCI-HBc-3A and pCI-HBc-3E expressing core protein with substitution of three major phosphorylation acceptor residues (serines) at the CTD by alanine (A) and aspartic acid (E), respectively, were provided by Dr. Jianming Hu at Pennsylvania State University [72]. The pHBV1.3-derived plasmids encoding single amino acid substituted Cp and pCMV-HBc-derived plasmids expressing single amino acid substituted Cp were generated by overlapping PCR strategy [32,54]. The plasmid expressing a full-length precore protein (p25) was constructed by insertion of precore coding region (nt 1816-nt 2454, genotype D, ayw) amplified with primers P25F and P25R (S2 Table) into the B*spD*I and P*st*I restricted pXF3H [73] and designated as pXF3H-p25-WT. The plasmid expressing p25 with substitution of Cp starting codon with AUA and/or insertion of a HA epitope tag at the immediate upstream of Cp starting codon was generated by Q5 Site-Directed Mutagenesis Kit (NEB, MA, USA, Cat. No. E0554) with pXF3H-p25-WT as a template and primers specified in S2 Table. The resulting plasmids were designated as pXF3H-p25_AUA_, pXF3H-p25/HA and pXF3H-p25HA, respectively (Fig 5A). To generate plasmids expressing p25HA with substitution of three major phosphorylation acceptor residues at the CTD of Cp, the CTD-coding region was amplified by PCR with pCI-HBc-3A or pCI-HBc-3E as a template. The 118 bp PCR product was then used as primer and pXF3H-p25HA as the template for second-round long fragment PCR. After digestion of input plasmids with D*pn*I (NEB, Cat. No. R0176S) for 30 min at 37°C, the PCR products were transformed into DH5α competent cells (NEB, Cat. No. C29871) and the resulting plasmids pXF3H-p25HA/3A and pXF3H-p25HA/3E were confirming by DNA sequencing. To generate plasmids expressing p25HA with substitution of all the seven phosphorylation acceptor residues at the CTD of Cp, cDNA with the specified mutations were synthesized at Integrated DNA Technologies IDT (MA, USA) (S3 Table) and cloned into *BspD*I and *Pst*I restricted pXF3H and yield plasmids pXF3H-p25HA/7A or pXF3H-p25HA/7E. To construct the plasmids expressing p25 with single amino acid substitution at P25, T33 or I105 of Cp, the entire p25 open reading frame was amplified by primes P25F and P25R from pHBV1.3-derived plasmids with the corresponding Cp mutation and subcloned into pXF3H. All the pXF3H-p25HA-derived plasmid expressing p25HA with single or double amino acid residue substitutions were generated by site-directed mutagenesis with the primers specified in S2 Table. The plasmids expressing HA-tagged p22 or p17, with or without single amino acid substitution, were constructed by PCR amplification of the corresponding coding region from pXF3H-p25HA and its derived plasmids with primers listed in S2 Table and subcloned the DNA fragment into pXF3H. All the plasmids generated were confirmed by DNA sequencing.

### Cell transfection and preparation of HBV virions

HepG2 cells were seeded into collagen-I rat tail (Corning, Cat. No. 354236)-coated plates or Petri dishes for 6 h before transfection. The cells were transfected with desired plasmid(s) with lipofectamine 2000 (Invitrogen, Cat. No. 11668–019) by following manufacturer’s direction. The culture media and cells will be harvested at the indicated time points post transfection for desired molecular analysis as specified below. To prepare HBV virion stocks for infection assays, the culture media of HepG2 cells transfected with pHBV1.3-WT or its derived plasmids encoding mutant Cp were harvested at 3, 6 and 9 days post transfection, respectively. Viral particles in the culture media were concentrated by 20% sucrose cushion ultracentrifugation at 27,000 rpm (Beckman, SW28) for 16 h at 4°C. The pellet was resuspended with desired volume of Opti-MEM (Gibco, Cat. No. 31985–070) (volume of Opti-MEM usually about 1% of culture media). Titers of HBV virions were determined by the IP-qPCR assay as described below.

### IP-qPCR quantification of secreted virions

One milliliter of supernatant from HepG2 cells transfected with pHBV1.3 or its derived plasmid encoding mutant Cp were precleared by addition of 20 μl Dynabeads Protein G beads (Invitrogen, Cat. No. 10004D) and incubated at 4°C for 60 min. After the removal of beads, anti-HBsAg antibody and anti-preS2 antibody were added at a ratio of 1:2 for a total of 6 μl into the pre-cleaned supernatant and incubated overnight at 4°C. Thirty microliters of Dynabeads Protein G beads were then added into each sample and incubated at 4°C for 4 h. The beads were washed with PBS (Corning, Cat. No. 21040-CMR) for 5 min per time for a total of 10 times. The beads were collected by magnetic rack and resuspended in core DNA lysis buffer (10 mM Tris-HCl, pH 8.0; 1 mM EDTA; 1% Nonidet P-40). Before extracted by phenol-chloroform, the samples were digested by DNase I (Promega, Cat. No. M610A) at 37°C for 30 min and followed by proteinase K (200 μg/mL) digestion at 45°C for 1 h. For quantification of HBV DNA, a SYBR Green Real-time PCR method were performed by Light-Cycler 480 II Real-time PCR Detection System (Roche, Mannheim, Germany). The primers used to detect HBV viral load is as follows: F (303–322 nt): 5′-TGGCCAAAATTCGCAGTCCC-3′, R (448–425 nt): 5′-GAAGAACCAACAAGAAGATGAGGC-3′ [74]. The serial dilutions of pHBV1.3 plasmid were used as standards of quantification.

### HBV infection of C3A^NTCP^ and PHHs

For C3A^NTCP^ cells infection, cells were seeded into collagen-I rat tail-coated plates for 24 h, and subjected to pretreat with DMEM supplemented with 3% FBS, 1% MEM NEAA (Gibco, Cat. No. 11140) and 2% DMSO for 24 h. The cells were then infected with HBV in DMEM containing 3% FBS, 2% DMSO, 1% MEM NEAA and 4% PEG-8000 (Sigma, Cat. No. P1458). The inoculums were removed at 16–24 h post infection (hpi) and the cell monolayers were washed with PBS for 5 times before refreshing with DMEM containing 3% FBS, 1% MEM NEAA and 2% DMSO. For PHHs infection, cells were maintained in HCM medium for 24 h, then infected with HBV in HCM containing 4% PEG-8000. The inoculums were removed at 16–24 hpi and the cell monolayers were washed with PBS for 5 times before refreshing with HCM.

### Extraction and detection of HBV DNA and RNA by hybridization and real time PCR assays

HBV capsid-associated (core) DNA and Hirt DNA were extracted from transfected HepG2 cells or HBV infected hepatocytes by following our published procedures [75,76]. Total cellular RNAs were extracted with Trizol reagent (Invitrogen, Grand island, NY) by following manufacturer’s directions. For extraction of encapsidated pgRNA, the cells were lysed by addition of 250 μl lysis buffer (10 mM Tris-HCl, pH 8.0; 1 mM EDTA; 1% Nonidet P-40) per well of 12-well plates and incubated in room temperature (RT) for 10 min. The cell lysates were centrifugated at 10,000 *g* for 10 min at 4°C. The supernatant was mixed with 6 Units of micrococcal nuclease (NEB, MA, USA) and 15 μl 100 mM CaCl_2_ and incubated at 37°C for 15 min. The reaction was terminated by addition of 6 μl of 0.5 M EDTA [77]. RNA in the reaction was extracted by adding the 750 μl Trizol LS reagent (Invitrogen Grand island, NY) by following manufacturer’s directions.

Southern blot and Northern blot detection of HBV DNA and RNA were described previously [78,79]. For detection of cccDNA, four-fifth of extracted Hirt DNA was denatured at 88°C for 8 minutes and chilled in ice for 5 min. The DNA were then digested by E*coR*I at 37°C for 45–60 min and subjected for Southern blot detection of HBV cccDNA as well as mitochondrial DNA (mtDNA). The remaining one-fifth of Hirt DNA was mixed with 5 μl 2×NEB 3 buffer, 1 μl T5 exonuclease (NEB, MA, USA) and nuclease-free water for a total of 10 μl and incubated at 37°C for 30 min. The reaction was terminated by incubation at 95°C for 5 min and subjected for quantification of HBV cccDNA by real time PCR. Specifically, the reaction mixture (20 μl) contained 1 μl of forward primer (10 μM) (5′-GCCTATTGATTGGAAAGTATGT-3′), 1 μl of reverse primer(5′-AGCTGAGGCGGTATCTA-3′) (10 μM), 4 μl of cccDNA template, 10 μl of 2× mix LightCycler 480 SYBR green Master Roche), and 4 μl of nuclease-free water. The reaction mixture was denatured at 95°C for 5 minutes, followed by 45 cycles at 95°C for 30 seconds, 60°C for 30 seconds, and 72°C for 30 seconds, 88°C for 2 seconds [80]. The serial dilutions of pHBV1.3 mer plasmid were used as standards of quantification.

### In vitro nucleocapsid uncoating assay

HepG2 cells cultured in 10 cm in diameter Petri dish were transfected with indicated plasmids. At 48 h post transfection, the cells were lysed with 2 ml core DNA lysis buffer per dish on ice for 15–20 min. The cell lysates were centrifugated at 16,000 *g* for 10 min at 4°C. The supernatant was overlayed onto 3 ml of 20% sucrose cushion and centrifugated at 46,000 rpm (Beckman, SW55) for 3.5 h at 4°C. The pellet was resuspended in 200 μl TNE buffer (0.15 M NaCl; 0.01M Tris-HCl, pH 7.4; 0.1 mM EDTA). Forty microliters of each capsid preparation were mixed with 50 μl of 2 × reaction buffer containing 0.3M NaCl, 0.1M Tris-HCl (pH 8.0), 20 mM MgCl_2_, 2 mM dithiothreitol, 0.2% (vol/vol) Nonidet P-40 and 0.2 mM each of dNTPs. GLS4 was then added into the indicated reactions to a final concentration of 1 μM. Nuclease-free water was added to bring the reaction volume to 100 μl. After incubation at 37°C for 16 h, the reaction was subjected for extraction of viral DNA with or without prior DNase I digestion at 37°C for 30 min. The DNA were resolved by 1.5% agarose gel electrophoresis and transferred onto Hybond-XL membrane. The membrane was probed with α-^32^P-UTP labeled minus strand specific full-length HBV riboprobe.

### Capsid electrophoresis mobility shift assay

HepG2 cells cultured in a 10 cm in diameter Petri dish were transfected with pCMV-HBc or its derived plasmid encoding Cp with a desired single amino substitution [32]. At 48 h post transfection, the cells were lysed with 2 ml core DNA lysis buffer for 10 minutes at RT. The cell lysates were centrifugated at 16,000 g for 10 min at 4°C. The supernatant was overlayed onto 3 ml of 20% sucrose cushion and centrifugated at 46,000 rpm (Beckman, SW55) for 3.5 h at 4°C. The pellet was resuspended in 200 μl of TNE buffer (10 mM Tris-HCl, pH 7.5, 150 mM NaCl, and 1 mM EDTA). Forty microliters of the capsid preparation were mixed with 50 μl of 2 × reaction buffer containing 0.3M NaCl, 0.1M Tris-HCl (pH 7.6), 20 mM MgCl_2_, 2 mM dithiothreitol, 0.2% (vol/vol) Nonidet P-40. GLS4 was then added into the indicated reactions to a final concentration of 2 μM. Nuclease-free water was added to bring the reaction volume to 100 μl. After incubation at 37°C for 6 h, 20 μl of the reaction was resolved in 1.8% agarose gel electrophoresis. The capsids were transferred onto a nitrocellulose membrane by soaking with TNE buffer. HBV capsids on the membrane were detected with a particle gels assay procedure described previously [81].

### Western blot assays

Cells per well of 12-well plate were lysed in 250 μl of 1 × LDS buffer (Invitrogen, Cat. No. NP0007) with or without addition of 2.5% 2-mercaptoethanol (BME) (Sigma) at RT for 10 min. The lysed samples were incubated at 95°C for 20 min. A aliquot of 20 μl of the cell lysate was resolved in a NuPAGE 12% Bis-Tris Protein Gel (Invitrogen) with NuPAGE MES SDS running buffer (Invitrogen) for SDS-PAGE and transferred onto PVDF membrane (Invitrogen) by using iBlot 2 dry blotting system (Thermo fisher scientific). The membrane was blocked with 5% nonfat milk in TBST (Tris-buffered saline containing 0.1% Tween 20) at RT for 1–2 h and probed with a desired primary antibody. Bounded antibody was revealed either by HRP-linked anti-rabbit/mouse IgG secondary antibodies and visualized by ChemiDOC Touch Image System (BioRad) or by Li-Cor IRDye goat anti-rabbit/mouse secondary antibody and visualized by Li-Cor Odyssey system (Li-Cor biotechnology).

### Phos-tag gel assay

SuperSep Phos-tag gel containing Phos-tag with zinc ion was purchased from Fujifilm Wako Cure Chemical Corporation (Cat. No. 199–18011). Denatured protein samples were resolved by electrophoresis with Novex Tris-Glycine SDS running buffer (Thermo fisher scientific). The gel was then gently soaked in transfer buffer containing 10mM EDTA for 20min for three times and followed by soaking the gel with transfer buffer without EDTA for 10min. Proteins in the gel were then transferred onto PVDF membrane. The membrane was blocked with 5% nonfat milk in TBST (Tris-buffered saline containing 0.1% Tween 20) at RT for 1–2 h. HBV precore were detected by probing the membrane with antibody HBc-170A. Bounded antibody was revealed by HRP-linked anti-rabbit IgG (CST, Cat. No. 7074S) secondary antibody and visualized by ChemiDOC Touch Image System (BioRad).

### IP-Western blot analysis of secreted HBeAg

One milliliter supernatant from transfected cells were incubated with 25 μl Pierce Anti-HA Magnetic Beads (Thermo scientific, Cat. No. 88836) at RT for 45 min. The beads were captured with magnetic rack and washed with Pierce IP lysis buffer (Thermo scientific, Cat. No. 87788) for 3 ×5 times followed by dissolving into 1 ×LDS buffer with or without addition of 2.5% BME, followed by boiled at 95°C for 20 min, after cooling on ice, samples were subjected to resolve into NuPAGE 12% Bis-Tris Protein Gel for SDS-PAGE. HBeAg was detected by anti-HA rabbit polyclonal antibodies.

### ELISA detection of HBsAg and HBeAg

HBsAg and HBeAg were detected by commercial HBsAg (Autobio, Cat. No. CL0310-2) and HBeAg (Autobio, Cat. No. CL0312-2) ELISA CLIA kit according to manufacturer’s instruction.

### Protein phosphatase treatment

HepG2 cells transfected with desired plasmids were lysed by core DNA lysis buffer, 40 μl lysed samples were incubated with 1μl Lambda phosphatase (NEB, MA, USA), 5 μl 10 ×NEB buffer 3.1 and 4 μl nuclease-free water for 40 min at 30°C. After incubation, samples were denatured by mixing with 1× LDS buffer containing 2.5% BME and boiled at 95°C for 20 min. After cooling on ice, samples were subjected to Western blot assay.

### Subcellular fractionation assay

Cytoplasmic, membrane and nuclear fractionations were all performed by using the Qproteome cell compartment kit (Qiagen, Cat. No. 37502) according to the manufacturer’s instructions. Briefly, HepG2 cells in 10 cm in diameter Petri dish were lysed and suspended with ice-cold lysis buffer for 10 min at 4°C on end-over-end shaker followed by centrifugating the lysate at 1000 *g* for 10 min at 4°C. The supernatant was harvested as cytosolic fraction. The pellet was lysed with Extraction buffer CE2 for 30 min at 4°C on end-over-end shaker, followed by centrifugating the lysate at 6000 *g* for 10 min at 4°C. The supernatant was harvested as membrane associated fraction. The pellet was treated with 7 μl Benzonase Nuclease and 13 μl distilled water for 15 min at RT, followed by suspending with Extraction buffer CE3 for 10 min at 4°C on end-over-end shaker, centrifuge the lysate at 6800 *g* for 10 min at 4°C. The supernatant was harvested as nuclear fraction. Mix the indicated fractions with final concentration of 1×LDS buffer containing 2.5% BME and denature at 95°C for 20 min. After cooling on ice, samples were subjected to Western blot assay.

### Statistical analysis

Statistical analysis was performed by unpaired student *t* test using Prism software (GraphPad). A value of *P* < 0.05 was considered statistically significant. ns: no significance; *:*P<0*.*05;* **: *P<0*.*01;* ***: *P<0*.*001*.

## Supporting information

S1 Fig Illustration of the domain structures of Cp and precore-derived proteins. Amino acid sequences of propeptide in precore region and arginine-rich C-terminal domain (ARD) are provided. The three major (red) and four minor (orange) phosphor-acceptor residues in the CTD are highlighted.
(TIF)

S2 Fig Substitution of Cp residue P25, T33 or I105 interferes pgRNA encapsidation. HepG2 cells were transfected with pHBV1.3 or derived plasmid encoding Cp with the indicated single amino acid substitution. Starting at 6 h post transfection, the cells were cultured in the absence or presence of 1μM Entecavir (ETV) and harvested at 72 h. Intracellular encapsidated pgRNA and total HBV RNA were analyzed by Northern blot hybridization with an α-^32^P-UTP labeled full-length minus-strand HBV RNA probe. The gray value of encapsidated pgRNA was quantified by Image J and presented as the percentage of that in cells transfected with WT HBV replicon in the presence of ETV treatment.
(TIF)

S3 Fig Substitution of Cp residues P25, T33 or I105 reduces the yield of virions. HepG2 cells were transfected with pHBV1.3 or a derived plasmid encoding Cp with the indicated single amino acid substitution and harvested at 72 h post transfection. Virions in culture media were immunoprecipitated with antibodies recognizing epitopes in S and pre-S2 regions of envelope proteins and virion DNA was quantified by qPCR (IP-qPCR assay). The serial dilutions of pHBV1.3 plasmid were used as standards of absolute quantification. The yields of HBV virions were presented as copies of virion DNA per milliliter of culture medium. The data (Mean ± SD) from three independent experiments were analyzed by two-tailed Student’s t-test (unpaired), ns: no significance; **: *P* < 0.01; ***: *P* < 0.001.
(TIF)

S4 Fig Substitution of Cp residue P25, T33 or I105 impairs virion infectivity. Hirt DNA was extracted from HBV infected C3A^NTCP^ cells described in the experiment presented in Fig 2C. Hirt DNA were denatured at 88°C for 8 min and restricted by E*coR*I to convert cccDNA into a unit-length double stranded linear DNA and detected by Southern blot hybridization with a riboprobe specifically hybridizing to negative strand DNA. Unit-length HBV linear DNA served as a molecular weight marker. mtDNA: mitochondria DNA.
(TIF)

S5 Fig Substitution of Cp residue P25, T33 or I105 promotes the secretion of virions with immature viral DNA species. HepG2 cells were transfected with pHBV1.3 or derived plasmid encoding Cp with the indicated single amino acid substitution. A total of 32 ml media were harvested at day 3 post transfection. Viral particles were concentrated by 20% sucrose ultracentrifugation for 27,000 rpm (Beckman, SW28) for 16 h and suspended with 300 μl Opti-MEM. Virion particles were immunoprecipitated with antibodies recognizing pre-S2 and S regions of envelope proteins. Virion DNA was quantified by a real-time PCR assay. Equal amounts of virion DNA from the different samples were resolved by 1.5% agarose gel and detected by Southern blot assay. RC, relaxed circular DNA. SS, single stranded DNA.
(TIF)

S6 Fig Differential subcellular distribution of phosphorylated and dephosphorylated p22 proteins. **(A)** HepG2 cells were transfected with pXF3H-p25HA or pXF3H-p22HA. Thirty-six hours post transfection, the cells were re-seeded onto round coverslips in the well of 24-well plates and cultured for an additional 12 h. The cells were fixed with 95% methanol and 5% glacial acetic acid, followed by incubation in blocking solution (5% BSA, 10% FBS and 0.3% Triton X-100) for 60 min. Intracellular p22 was detected by HA tag antibody. Cell nuclei were counterstained with DAPI. Images were captured by microscopy using a 60× objective. Scale bar: 10 μM. (**B**) HepG2 cells were transfected with pXF3H-p25HA or pXF3H-p22HA and harvested at 48 h post transfection. The cytosol, nuclear and membrane associated fractions were prepared by Qiagen cell compartment kit according to the manufacturer’s instructions. Precore-derived proteins in each of the fractions were detected by Western blot assay with anti-HBc-170A. Calnexin, α-tubulin and Lamin A/C were detected by the corresponding antibodies.
(TIF)

S7 Fig Unphosphorylated p22, but not the phosphorylated p22, is the precursor of HBeAg. **(A)** HepG2 cells were transfected with pXF3H-p25HA expressing WT precore, cells were mock (DMSO)-treated or treated with furin inhibitor (2 μM) starting at the time of transfection for 48 h. HBeAg in culture media were measured by CLIA. Intracellular p22 were detected by Western blot assays with antibody against HA tag. β-actin served as a loading control. The levels of unphosphorylated p22 were determined by ChemiDOC Touch Image System (BioRad) and normalized to β-actin. The results (mean ± SD) from a biologically triplicate experiment are presented. Data were analyzed by two-tailed Student’s t-test (unpaired), **: *P* < 0.01. (**B**) HepG2 cells were transfected with pXF3H-p25HA and pXF3H-p22HA derived plasmid expressing WT precore and harvested at 48 h post transfection. Intracellular p22 was detected by Western blot assays with antibody against HA tag. β-actin served as a loading control. Secreted p17 was detected by IP-Western blot assay. HBeAg in culture media were measured by CLIA kit. Result for Western blot was shown as one representative image. Result (mean ± SD) for HBeAg levels from three independent experiments were analyzed by two-tailed Student’s t-test (unpaired). ***: *P* < 0.001.
(TIF)

S8 Fig Intramolecular and intermolecular disulfide bond formation in precore protein biogenesis. HepG2 cells were transfected with pXF3H-p25HA_ΔCTD_ or derived plasmid expressing WT p17HA or p17HA-C(-7)A and harvested at 48 h post transfection. The secreted p17 was concentrated by immunoprecipitation. Iodoacetamide (IAM) was added into culture media to a final concentration of 50 μM to prevent disulfide bond formation during IP procedure. Cells or eluted pellet were lysed by LDS buffer with or without BME addition. Intracellular (**A**) and secreted (**B**) p17 were detected by Western blot assay with an antibody against HA tag.
(TIF)

S9 Fig GLS4 did not apparently accelerate the decay of intracellular p17. **(A)** HepG2 cells were transfected with pXF3H-p25HA_ΔCTD_ expressing p17HA. At 36 h post transfection, the cells were cultured with media containing 50 μg/ml puromycin, 50 μg/ml cycloheximide (CHX) without or with 1 μM GLS4 for 12 h. The cells were harvested at the indicated time points. Intracellular p17 were detected by Western blot assay with an antibody against HA tag. The efficient arrest of protein biosynthesis by CHX was monitored by Western blot detection of incorporated puromycin. β-actin served as a loading control. **(B)** The level of p17 protein signal at each time point in panel A were quantified by Photoshop and normalized to β-actin and plotted as the fraction of p17 level at the starting point (0 h) of protein synthesis arrest by CHX. Data (mean ± SD) from three independent experiments are plotted and analyzed by two-tailed Student’s t-test (unpaired). ns: no significance.
(TIF)

S10 Fig Inhibition of the proteolytic activities of proteasomes and/or lysosomes did not apparently alter the levels of intracellular p17. HepG2 cells were transfected with pXF3H-p25HA_ΔCTD_ or derived plasmid expressing WT or the indicated mutant p17HA. At 36 h post transfection, the cells were mock (DMSO)-treated or treated with 50 μM MG132, 50 μM chloroquine (CQ) alone or in combination for 10 h. Intracellular p17 was detected by Western blot assay with an antibody against HA. β-actin served as a loading control. LC3A/B served as a marker for the inhibition of autophagy flux by CQ. Accumulation of ubiquitinated proteins served a marker of efficient inhibition of proteasome activity by MG132.
(TIF)

S1 Table Antiviral activity of GLS4 against WT HBV and Cp mutant HBV variants in HepG2 cells. (DOCX)

S2 Table Sequence of the primers for plasmid construction. (DOCX)

S3 Table Sequence of synthesized 7A and 7E cDNA. (DOCX)

S4 Table Information of antibodies used. (DOCX)

S1 Data Excel spreadsheet containing, in separate sheets, the underlying numerical data and statistical analysis for Figure panels: Figs 2C–2F, 3C, 3D, 4A–4C, 5B, 8A–8D, 9, 10B, 10C, S3A, S7A, S7B and S9. (XLSX)
