# Supplementary figures and images for: Amino acid residues at core protein dimer-dimer interface modulate multiple steps of hepatitis B virus replication and HBeAg biogenesis
Source: PLoS Pathog. 2021 Nov 9;17(11):e1010057. doi: 10.1371/journal.ppat.1010057 (PMC8604296; doi:10.1371/journal.ppat.1010057)

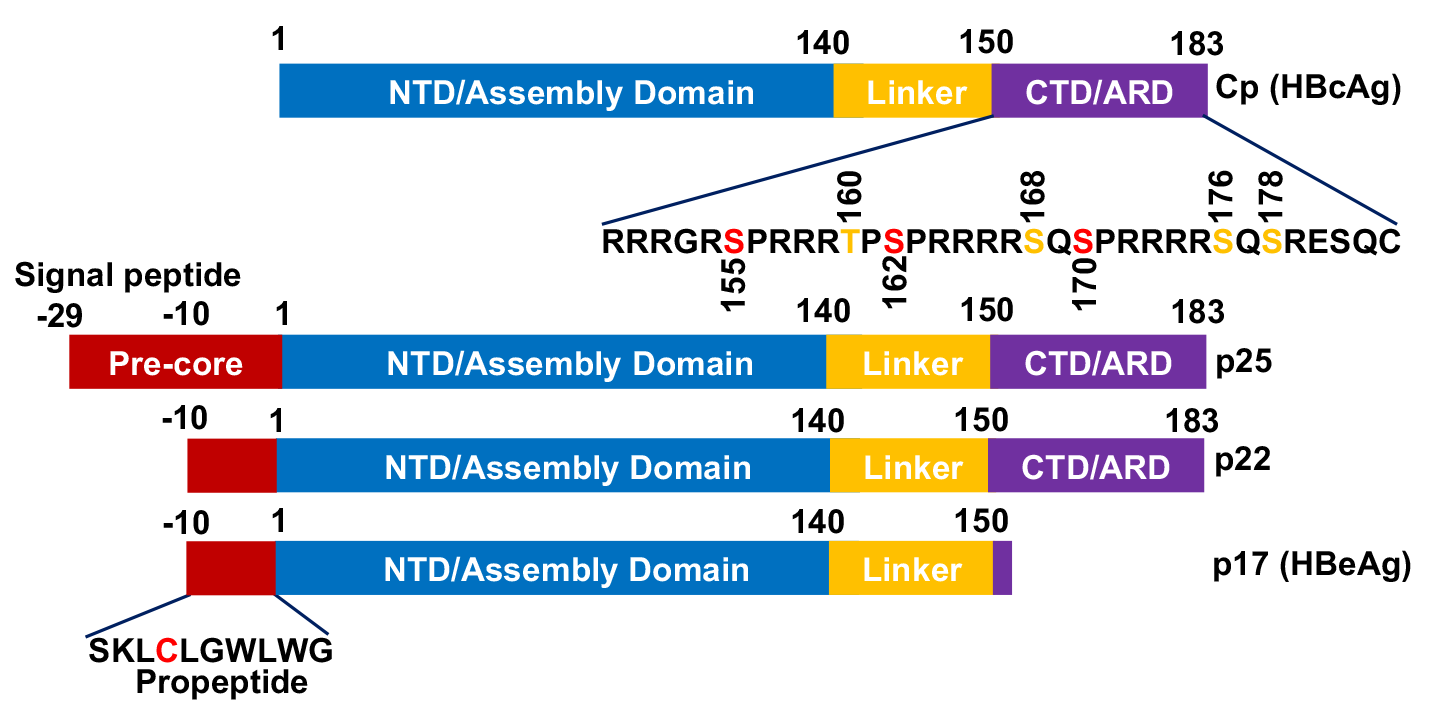

Supplement: S1 Fig — Amino acid sequences of propeptide in precore region and arginine-rich C-terminal domain (ARD) are provided. The three major (red) and four minor (orange) phosphor-acceptor residues in the CTD are highlighted. (TIF) [file ppat.1010057.s001.tif]

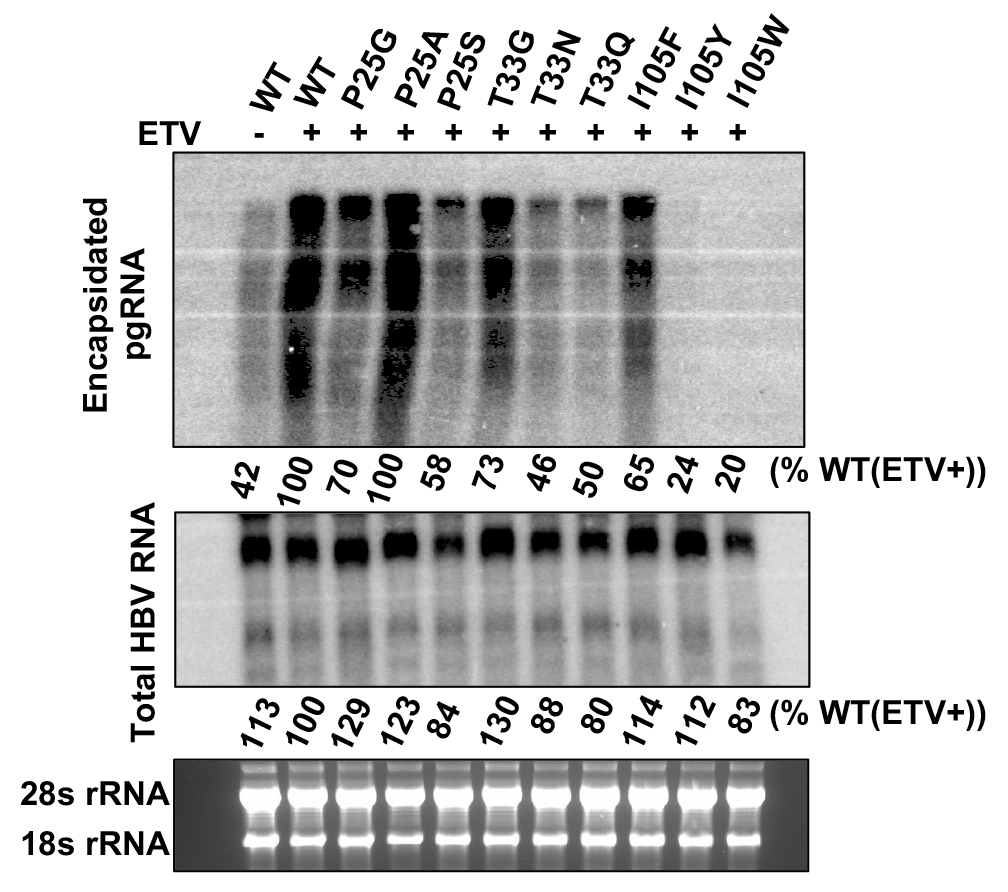

Supplement: S2 Fig — HepG2 cells were transfected with pHBV1.3 or derived plasmid encoding Cp with the indicated single amino acid substitution. Starting at 6 h post transfection, the cells were cultured in the absence or presence of 1μM Entecavir (ETV) and harvested at 72 h. Intracellular encapsidated pgRNA and total HBV RNA were analyzed by Northern blot hybridization with an α-32P-UTP labeled full-length minus-strand HBV RNA probe. The gray value of encapsidated pgRNA was quantified by Image J and presented as the percentage of that in cells transfected with WT HBV replicon in the presence of ETV treatment. (TIF) [file ppat.1010057.s002.tif]

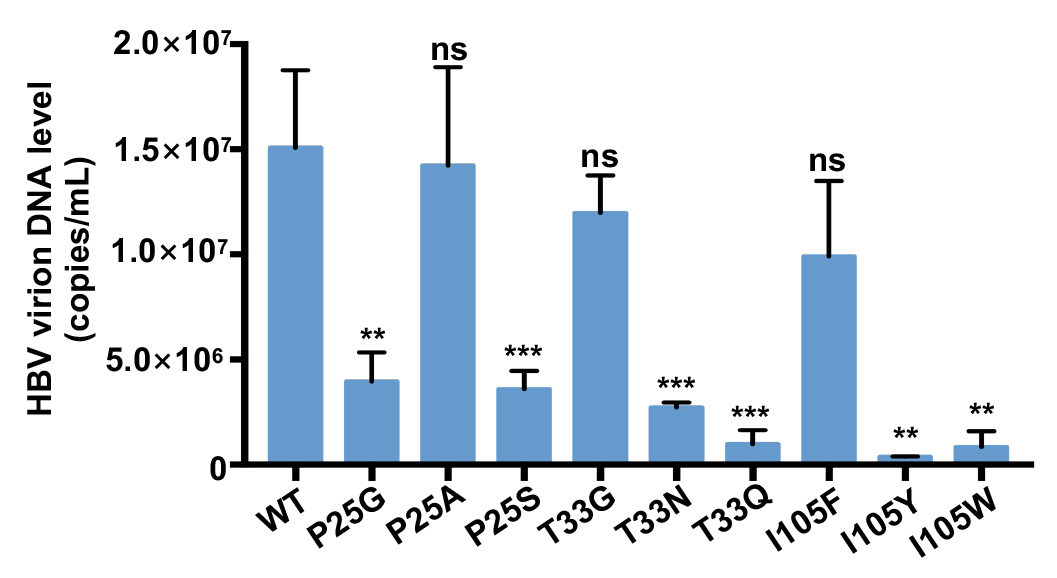

Supplement: S3 Fig — HepG2 cells were transfected with pHBV1.3 or a derived plasmid encoding Cp with the indicated single amino acid substitution and harvested at 72 h post transfection. Virions in culture media were immunoprecipitated with antibodies recognizing epitopes in S and pre-S2 regions of envelope proteins and virion DNA was quantified by qPCR (IP-qPCR assay). The serial dilutions of pHBV1.3 plasmid were used as standards of absolute quantification. The yields of HBV virions were presented as copies of virion DNA per milliliter of culture medium. The data (Mean ± SD) from three independent experiments were analyzed by two-tailed Student’s t-test (unpaired), ns: no significance; **: P < 0.01; ***: P < 0.001. (TIF) [file ppat.1010057.s003.tif]

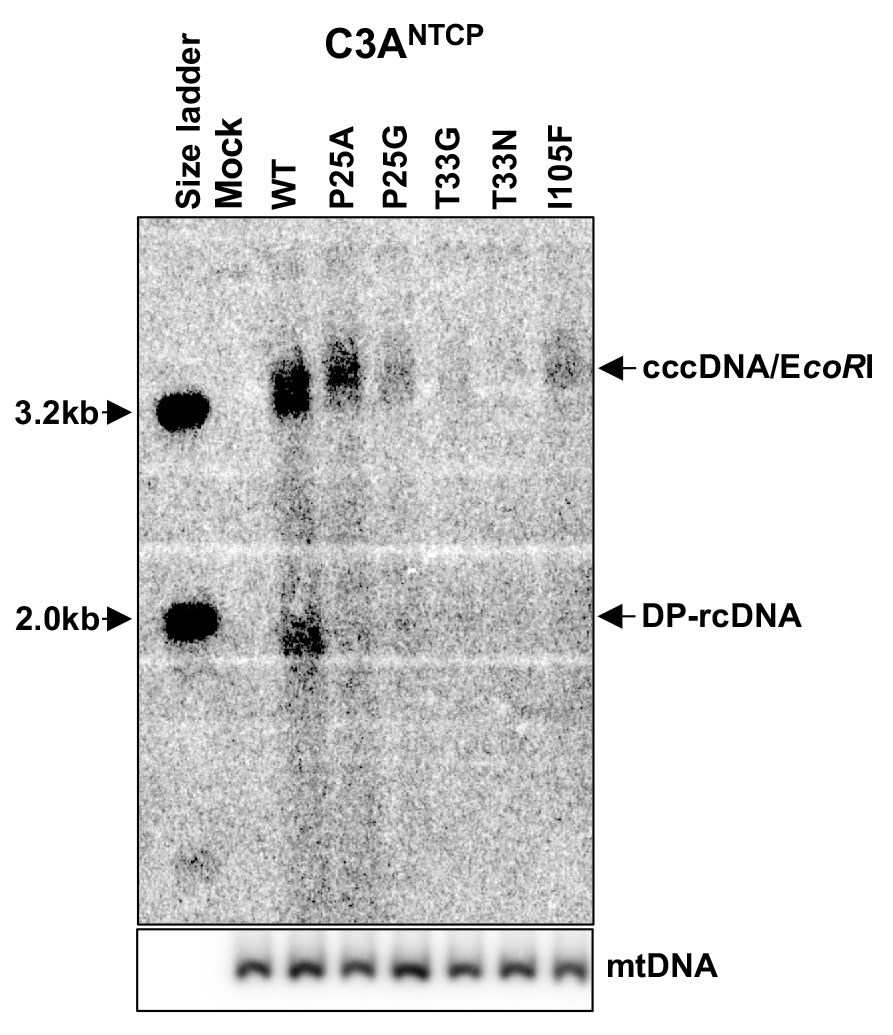

Supplement: S4 Fig — Hirt DNA was extracted from HBV infected C3ANTCP cells described in the experiment presented in Fig 2C. Hirt DNA were denatured at 88°C for 8 min and restricted by EcoRI to convert cccDNA into a unit-length double stranded linear DNA and detected by Southern blot hybridization with a riboprobe specifically hybridizing to negative strand DNA. Unit-length HBV linear DNA served as a molecular weight marker. mtDNA: mitochondria DNA. (TIF) [file ppat.1010057.s004.tif]

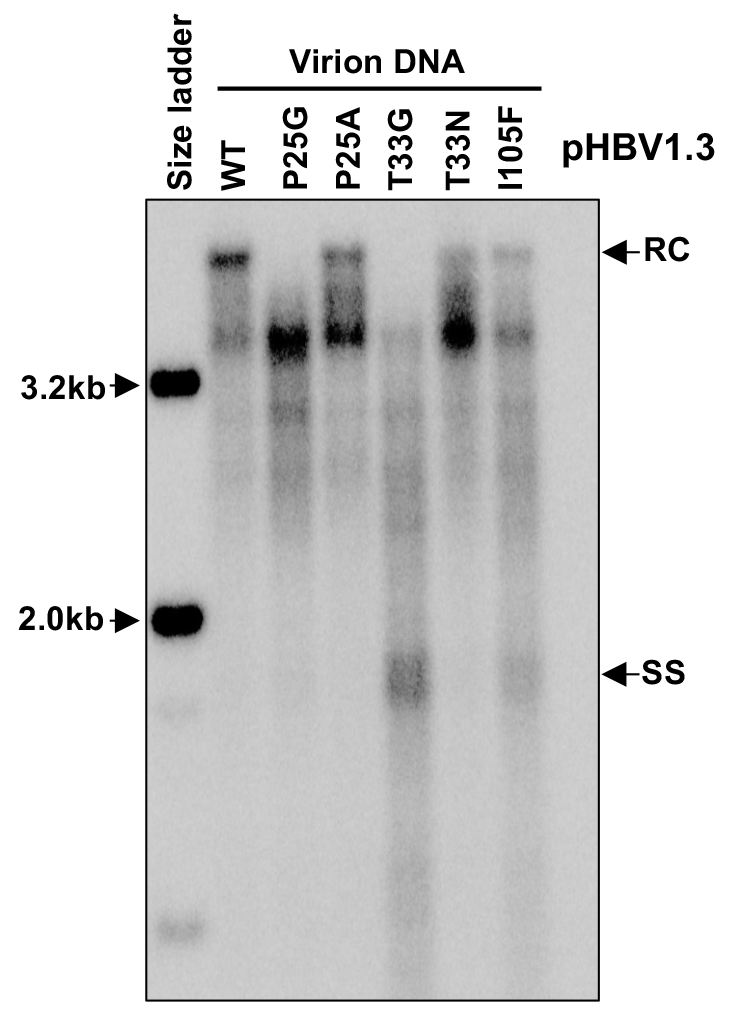

Supplement: S5 Fig — HepG2 cells were transfected with pHBV1.3 or derived plasmid encoding Cp with the indicated single amino acid substitution. A total of 32 ml media were harvested at day 3 post transfection. Viral particles were concentrated by 20% sucrose ultracentrifugation for 27,000 rpm (Beckman, SW28) for 16 h and suspended with 300 μl Opti-MEM. Virion particles were immunoprecipitated with antibodies recognizing pre-S2 and S regions of envelope proteins. Virion DNA was quantified by a real-time PCR assay. Equal amounts of virion DNA from the different samples were resolved by 1.5% agarose gel and detected by Southern blot assay. RC, relaxed circular DNA. SS, single stranded DNA. (TIF) [file ppat.1010057.s005.tif]

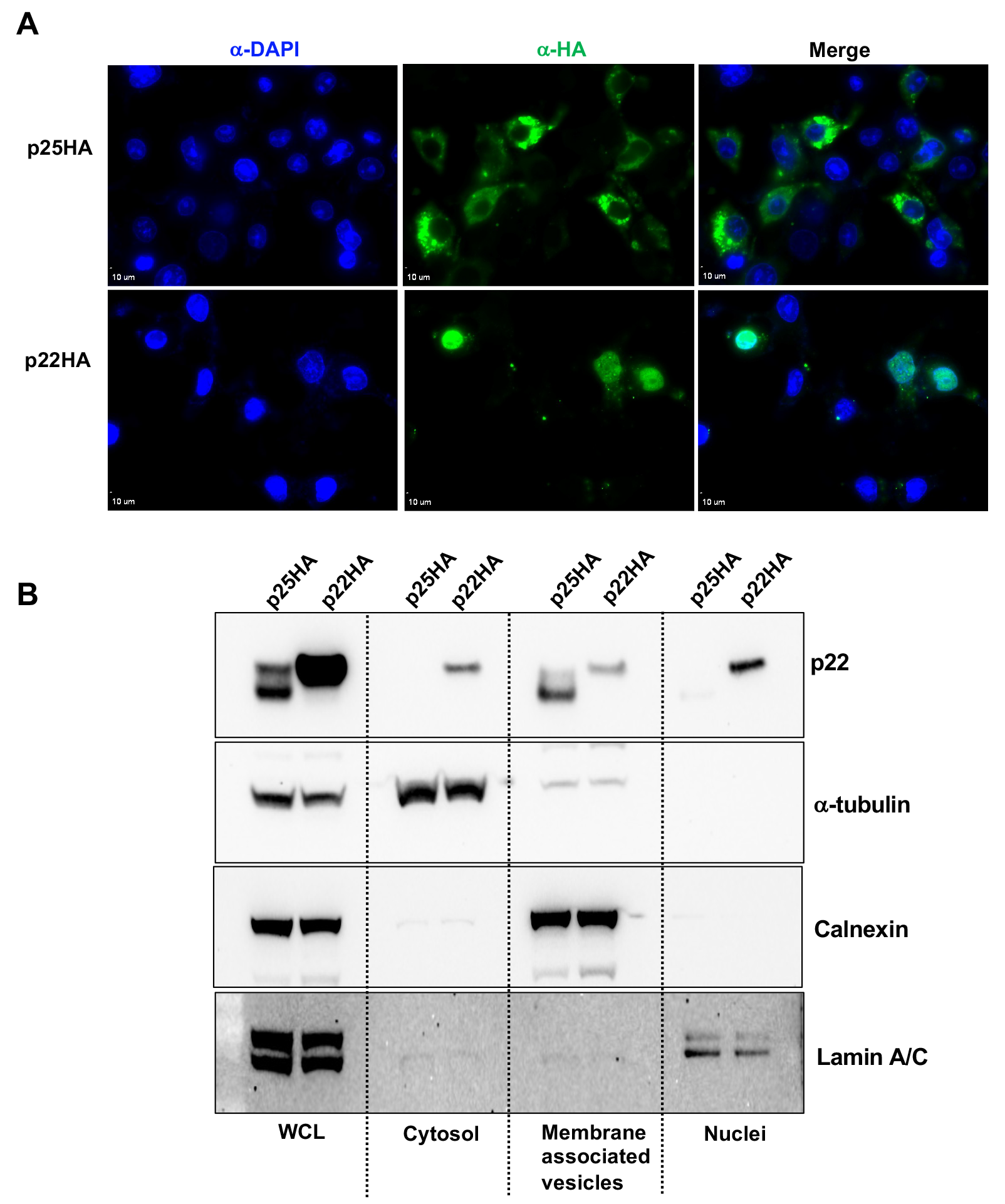

Supplement: S6 Fig — (A) HepG2 cells were transfected with pXF3H-p25HA or pXF3H-p22HA. Thirty-six hours post transfection, the cells were re-seeded onto round coverslips in the well of 24-well plates and cultured for an additional 12 h. The cells were fixed with 95% methanol and 5% glacial acetic acid, followed by incubation in blocking solution (5% BSA, 10% FBS and 0.3% Triton X-100) for 60 min. Intracellular p22 was detected by HA tag antibody. Cell nuclei were counterstained with DAPI. Images were captured by microscopy using a 60× objective. Scale bar: 10 μM. (B) HepG2 cells were transfected with pXF3H-p25HA or pXF3H-p22HA and harvested at 48 h post transfection. The cytosol, nuclear and membrane associated fractions were prepared by Qiagen cell compartment kit according to the manufacturer’s instructions. Precore-derived proteins in each of the fractions were detected by Western blot assay with anti-HBc-170A. Calnexin, α-tubulin and Lamin A/C were detected by the corresponding antibodies. (TIF) [file ppat.1010057.s006.tif]

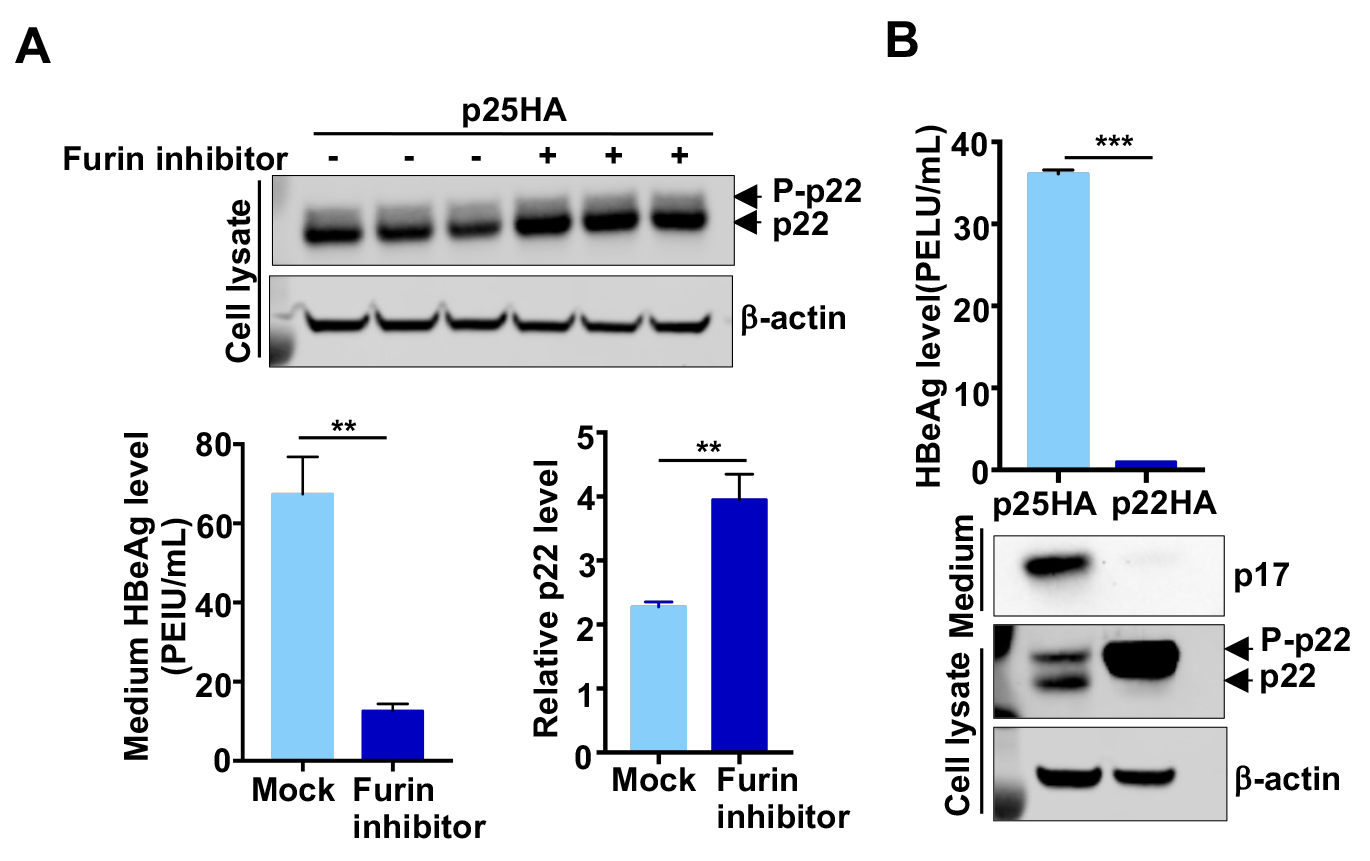

Supplement: S7 Fig — (A) HepG2 cells were transfected with pXF3H-p25HA expressing WT precore, cells were mock (DMSO)-treated or treated with furin inhibitor (2 μM) starting at the time of transfection for 48 h. HBeAg in culture media were measured by CLIA. Intracellular p22 were detected by Western blot assays with antibody against HA tag. β-actin served as a loading control. The levels of unphosphorylated p22 were determined by ChemiDOC Touch Image System (BioRad) and normalized to β-actin. The results (mean ± SD) from a biologically triplicate experiment are presented. Data were analyzed by two-tailed Student’s t-test (unpaired), **: P < 0.01. (B) HepG2 cells were transfected with pXF3H-p25HA and pXF3H-p22HA derived plasmid expressing WT precore and harvested at 48 h post transfection. Intracellular p22 was detected by Western blot assays with antibody against HA tag. β-actin served as a loading control. Secreted p17 was detected by IP-Western blot assay. HBeAg in culture media were measured by CLIA kit. Result for Western blot was shown as one representative image. Result (mean ± SD) for HBeAg levels from three independent experiments were analyzed by two-tailed Student’s t-test (unpaired). ***: P < 0.001. (TIF) [file ppat.1010057.s007.tif]

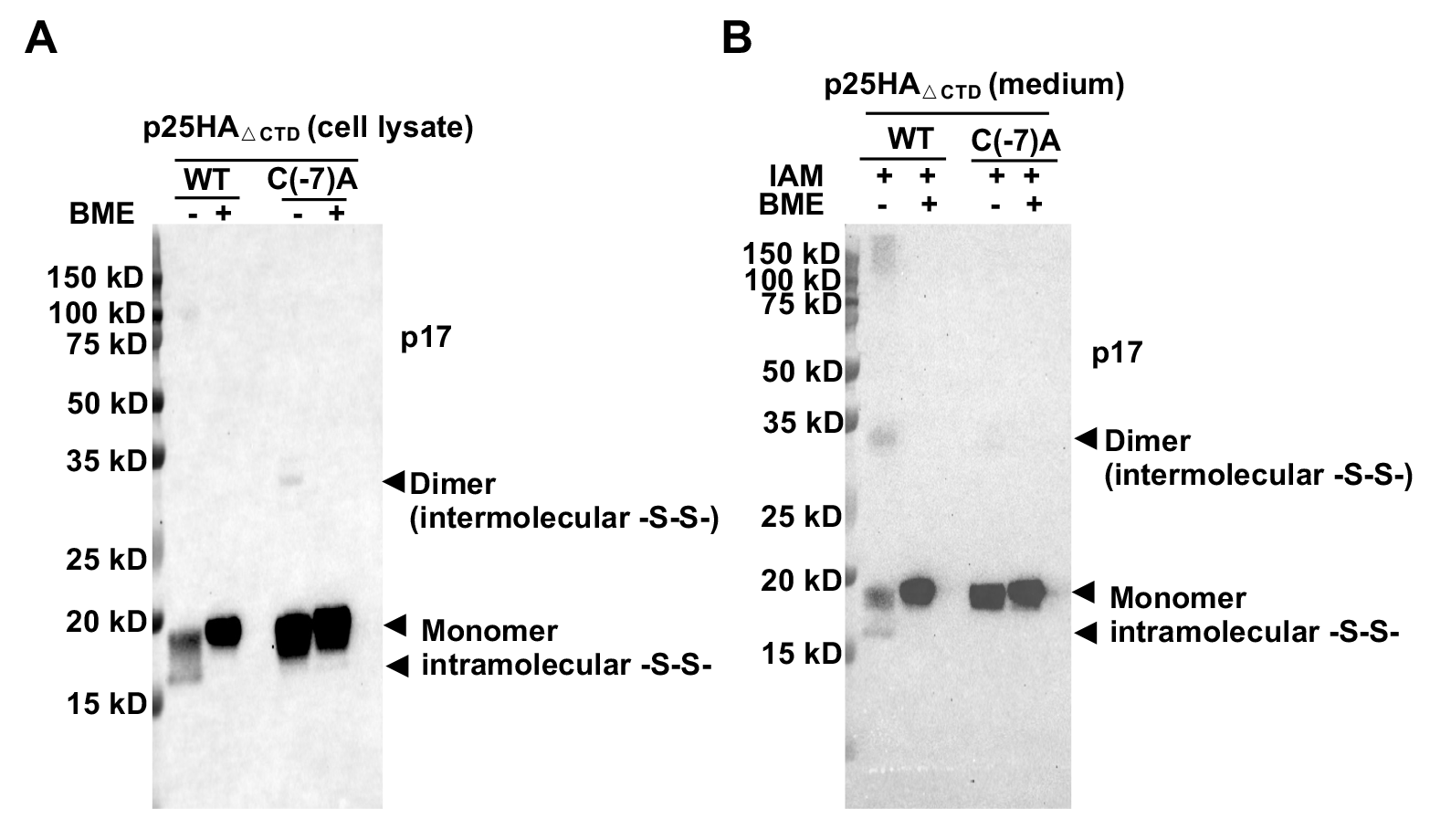

Supplement: S8 Fig — HepG2 cells were transfected with pXF3H-p25HAΔCTD or derived plasmid expressing WT p17HA or p17HA-C(-7)A and harvested at 48 h post transfection. The secreted p17 was concentrated by immunoprecipitation. Iodoacetamide (IAM) was added into culture media to a final concentration of 50 μM to prevent disulfide bond formation during IP procedure. Cells or eluted pellet were lysed by LDS buffer with or without BME addition. Intracellular (A) and secreted (B) p17 were detected by Western blot assay with an antibody against HA tag. (TIF) [file ppat.1010057.s008.tif]

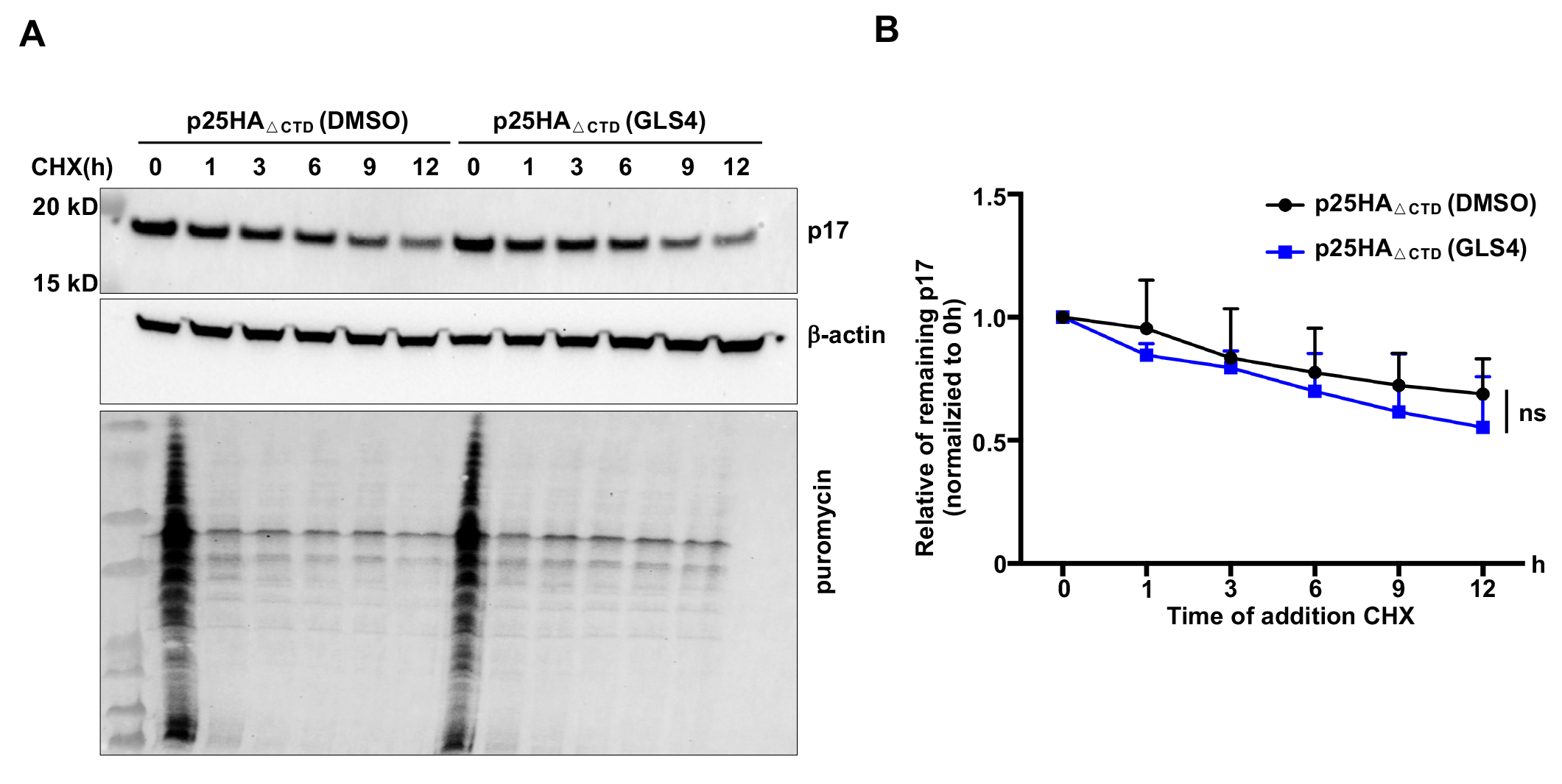

Supplement: S9 Fig — (A) HepG2 cells were transfected with pXF3H-p25HAΔCTD expressing p17HA. At 36 h post transfection, the cells were cultured with media containing 50 μg/ml puromycin, 50 μg/ml cycloheximide (CHX) without or with 1 μM GLS4 for 12 h. The cells were harvested at the indicated time points. Intracellular p17 were detected by Western blot assay with an antibody against HA tag. The efficient arrest of protein biosynthesis by CHX was monitored by Western blot detection of incorporated puromycin. β-actin served as a loading control. (B) The level of p17 protein signal at each time point in panel A were quantified by Photoshop and normalized to β-actin and plotted as the fraction of p17 level at the starting point (0 h) of protein synthesis arrest by CHX. Data (mean ± SD) from three independent experiments are plotted and analyzed by two-tailed Student’s t-test (unpaired). ns: no significance. (TIF) [file ppat.1010057.s009.tif]

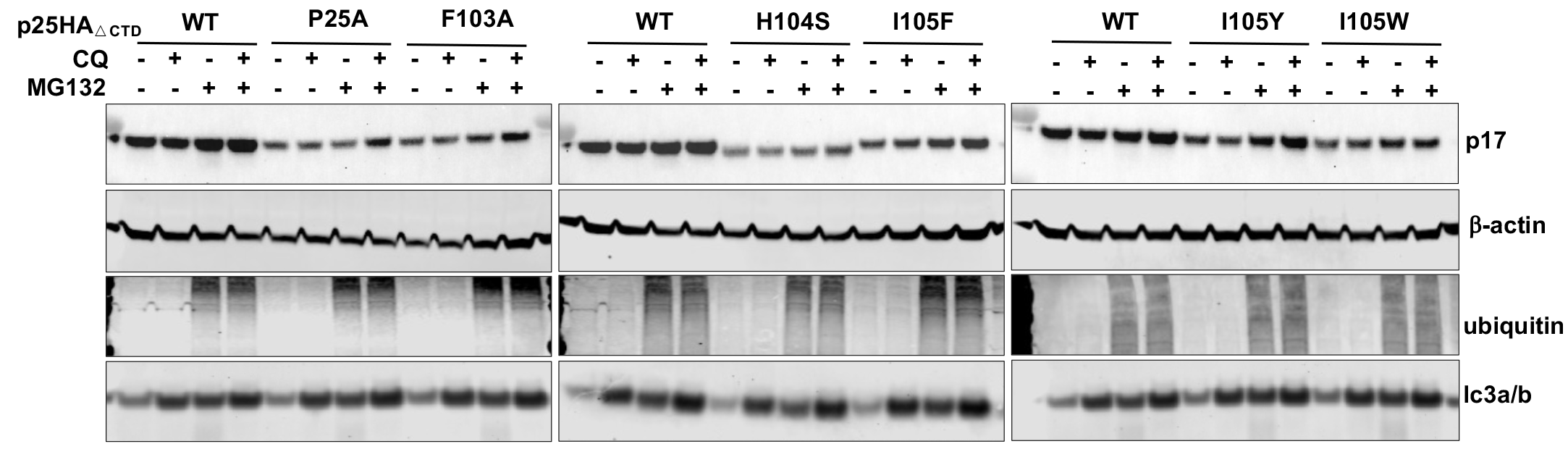

Supplement: S10 Fig — HepG2 cells were transfected with pXF3H-p25HAΔCTD or derived plasmid expressing WT or the indicated mutant p17HA. At 36 h post transfection, the cells were mock (DMSO)-treated or treated with 50 μM MG132, 50 μM chloroquine (CQ) alone or in combination for 10 h. Intracellular p17 was detected by Western blot assay with an antibody against HA. β-actin served as a loading control. LC3A/B served as a marker for the inhibition of autophagy flux by CQ. Accumulation of ubiquitinated proteins served a marker of efficient inhibition of proteasome activity by MG132. (TIF) [file ppat.1010057.s010.tif]
