# Supplementary material for: Amino acid residues at core protein dimer-dimer interface modulate multiple steps of hepatitis B virus replication and HBeAg biogenesis
Source: PLoS Pathog. 2021 Nov 9;17(11):e1010057. doi: 10.1371/journal.ppat.1010057 (PMC8604296; doi:10.1371/journal.ppat.1010057)
Supplement: S1 Table — (DOCX) [file ppat.1010057.s011.docx]

**S1 Table. Antiviral activity of GLS4 against WT HBV and Cp mutant HBV variants in HepG2 cells*.**

| pHBV1.3 | WT | P25G | P25A | T33G | T33N | T33Q | I105F | I105Y | I105W |
| --- | --- | --- | --- | --- | --- | --- | --- | --- | --- |
| EC_50_(nM) | 4.98 | >2000 | 1749 | >2000 | >2000 | >2000 | >2000 | >2000 | >2000 |
| EC_90_(nM) | 473.68 | >2000 | >2000 | >2000 | >2000 | >2000 | >2000 | >2000 | >2000 |

***Note:** HepG2 cells were transfected with pHBV1.3 or derived plasmid encoding Cp with the indicated single amino acid substitution. The cells were treated with a serial concentration of GLS4, starting at 6 h post transfection for 72 h. Intracellular core DNA were quantified by a qPCR assay. The concentration of GLS4 that reduces viral DNA by 50% (EC_50_) or 90% (EC_90_) was calculated from a biologically triplicate experiment by Prism software (GraphPad)**.**
