# Supplementary material for: Amino acid residues at core protein dimer-dimer interface modulate multiple steps of hepatitis B virus replication and HBeAg biogenesis
Source: PLoS Pathog. 2021 Nov 9;17(11):e1010057. doi: 10.1371/journal.ppat.1010057 (PMC8604296; doi:10.1371/journal.ppat.1010057)
Supplement: S2 Table — (DOCX) [file ppat.1010057.s012.docx]

**S2 Table. Sequence of the primers for plasmid construction.**

**____________________________________________________________________________**

| **plasmid** | **Primer Sequence (5’-3’)** |
| --- | --- |
| P25 | F: CGCATCGAT ATG CAACTT TTTCACCTCTGC C  R: AAAACTGCAGCTAACATTGAGG TTCCCGAG |
| P25_AUA_ | F: TGGGGCATAGACATCGACCCTTATAAA  R: AAGCCACCCAAGGCACAGC |
| P25/HA | F: ACGTTCCAGATTACGCTGGCATGGACATCGACCCTTA TAAA  R: CGTATGGGTAGATGTCCACGCCCCAAAGCCACCCAAGG |
| P25HA | F: ACGTTCCAGATTACGCTGGCATAGACATCGACCCTTATAAA  R: CGTATGGGTAGATGTCCACGCCCCAAAGCCACCCAAGG |
| P25HA-3A | Phos-F: CTGTTGTTAGACGACGAGGCAG  Phos-R: TGCAGCTAACATTGAGGTTCCCG |
| P25HA-3E | Phos-F: CTGTTGTTAGACGACGAGGCAG  Phos-R: TGCAGCTAACATTGAGGTTCCCG |
| P22HA | F: CGCATCGATATGTCCAAGCTGTGCCTTGGG  R: AAAACTGCAGCTAACATTGAGG TTC CCGAG |
| P22HA-3A | F: CGCATCGATATGTCCAAGCTGTGCCTTGGG  R: AAAACTGCAGCTAACATTGAGGTTCCCG |
| P22HA-7A | F: CGCATC GATATGTCCAAGCTGTGCCTTGGG  R: AAAACTGCAGCTAACATTGAGGTTCCCG |
| P17HA | F: ACGTTCCAGATTACGCTGGCATAGACATCGACCCTTATAAA  R: AAAACTGCAGCTACCTGCCTCGTCGTCTAACAA |
| P17HA/ P17HA -P25A/T33N /I105F/Y/W | F: ACGTTCCAGATTACGCTGGCATAGACATCGACCCTTATAAA  R: AAAACTGCAGCTACCTGCCTCGTCGTCTAACAA |
| P25HA-K (-9) D | F: CTCC GAT CTGTGCCTTGGG  R: GCTTGAACAGTAGGACATGAACAAG |
| P25HA-K (-9) A | F: CTCC GCG CTGTGCCTTGGG  R: GCTTGAACAGTAGGACATGAACAAG |
| P25HA-C (-7) A/ P17HA-C (-7) A | F: AAGCTGGCGCTTGGGTGGCTTTG  R: GGAGGCTTGAACAGTAGGACA |
| P25HA-C (-7) G | F:AAGCTGGGACTTGGGTGGCTTTG  R: GGAGGCTTGAACAGTAGGACA |
| P25HA-C (-7) S | F: AAGCTGAGCCTTGGGTGGCTTTG  R: GGAGGCTTGAACAGTAGGACA |
| P25HA-W (-4) L | F: CCTTGGG CTG CTTTGGGGCGTG  R: CACAGCTTGGAGGCTTGAACAG |
| P25HA-W (-2) L | F: GCTTCTGGGCGTGGACATCTAC  R: CACCCAAGGCACAGCTTGGA |
| P25HA-P45A | F: GTCTGCGGAGCATTGTTCACCTC  R: TCTAAGGCTTCCCGATACAGAGC |
| P25HA-P50A | F: TTCA GCG CACCATACTGCACTCAG  R: CAATGCTCAGGAGACTCTAAGGC |
| P25HA-C61A/ P17HA-C61A | F: ATTCTTGCGTGGGGGGAACTAATG  R: TGCTTGCCTGAGTGCAGTATGG |
| P25HA-C61G | F:ATTCTTGGATGGGGGGAACTAATG  R: TGCTTGCCTGAGTGCAGTATGG |
| P25HA-C61S | F:ATTCTTAGCTGGGGGGAACTAATG  R: TGCTTGCCTGAGTGCAGTATGG |
| P25HA-P79A | F: AGATGCGGCGTCTAGAGACCTAG  R: TCCAAATTAACACCCACCCAGGT |
| P25HA-Y88F | F: CAGTGCG GTCAACACTAATATGGGC  R: ACTACTAGGTCTCTAGACGCTGGAT |
| P25HA-Y88A | F: CAGTGCG GTCAACACTAATATGGGC  R: ACTACTAGGTCTCTAGACGCTGGAT |
| P25HA-K96D | F: CCTAGATTTCAGGCAACTCTTG  R: CCCATATTAGTGTTGACATAACTGA |
| P25HA-K96A | F: CCTAGCGTTCAGGCAACTCTTG  R: CCCATATTAGTGTTGACATAACTGA |
| P25HA-F97A | F: AAAGGCGAGGCAACTCTTGTGGTT  R: AGGCCCATATTAGTGTTGACATAAC |
| P25HA-K96A+F97A | F: CTAGCGGCGAGGCAACTCTTGTGGT  R: GCCCATATTAGTGTTGACATAACTG |
| P25HA-F103A/ P17HA-F103A | F: TGG GCTCACATTTCTTGTCTCACTT  R: CAAGAGTTGCCTGAACTTTAGGCC |
| P25HA-H104S/ P17HA-H104S | F: GGTTT TCC ATTTCTTGTCTCACTTT  R: ACAAGAGTTGCCTGAACTTTAGGC |
| P25HA-V124A | F: CGGAGCGTGGATTCGCACT  R: AAAGACACCAAATACTCTATAACTG |
| P25HA-I126A | F: GTGGGCGCGCACTCCTCCAG  R: ACTCCGAAAGACACCAAATACTCT |
| P25HA-P129A | F: CACTGCGCCAGCTTATAGACCACC  R: CGAATCCACACTCCGAAAGACAC |
| P25HA-S141A | F: CCTAGCGACACTTCCGGAGACTAC  R: ATAGGGGCATTTGGTGGTCTAT |
| P25HA-GG | F: GGCAACTTTTTCACCTCTGCCTAATCA  R: CATATCGATAGAACCGAGGTGCAG |
