# Supplementary material for: Amino acid residues at core protein dimer-dimer interface modulate multiple steps of hepatitis B virus replication and HBeAg biogenesis
Source: PLoS Pathog. 2021 Nov 9;17(11):e1010057. doi: 10.1371/journal.ppat.1010057 (PMC8604296; doi:10.1371/journal.ppat.1010057)
Supplement: S3 Table — (DOCX) [file ppat.1010057.s013.docx]

**S3 Table. Sequence of synthesized p22-7A and p22-7E cDNA.**

**______________________________________________________________________________**

**7A:**

CGCATCGATATGCAACTTTTTCACCTCTGCCTAATCATCTCTTGTTCATGTCCTACTGTTCAAGCCTCCAAGCTGTGCCTTGGGTGGCTTTGGGGCGTGGACATCTACCCATACGACGTTCCAGATTACGCTGGCATAGACATCGACCCTTATAAAGAATTTGGAGCTACTGTGGAGTTACTCTCGTTTTTGCCTTCTGACTTCTTTCCTTCAGTACGAGATCTTCTAGATACCGCCTCAGCTCTGTATCGGGAAGCCTTAGAGTCTCCTGAGCATTGTTCACCTCACCATACTGCACTCAGGCAAGCAATTCTTTGCTGGGGGGAACTAATGACTCTAGCTACCTGGGTGGGTGTTAATTTGGAAGATCCAGCGTCTAGAGACCTAGTAGTCAGTTATGTCAACACTAATATGGGCCTAAAGTTCAGGCAACTCTTGTGGTTTCACATTTCTTGTCTCACTTTTGGAAGAGAAACAGTTATAGAGTATTTGGTGTCTTTCGGAGTGTGGATTCGCACTCCTCCAGCTTATAGACCACCAAATGCCCCTATCCTATCAACACTTCCGGAGACTACTGTTGTTAGACGACGAGGCAGGGCCCCTAGAAGAAGAGCGCCCGCGCCTCGCAGACGAAGGGCACAAGCGCCGCGTCGCAGAAGAGCACAAGCACGGGAACCTCAATGTTAGCTGCAGAAAA

______________________________________________________________________________

**7E:**

CGCATCGATATGCAACTTTTTCACCTCTGCCTAATCATCTCTTGTTCATGTCCTACTGTTCAAGCCTCCAAGCTGTGCCTTGGGTGGCTTTGGGGCGTGGACATCTACCCATACGACGTTCCAGATTACGCTGGCATAGACATCGACCCTTATAAAGAATTTGGAGCTACTGTGGAGTTACTCTCGTTTTTGCCTTCTGACTTCTTTCCTTCAGTACGAGATCTTCTAGATACCGCCTCAGCTCTGTATCGGGAAGCCTTAGAGTCTCCTGAGCATTGTTCACCTCACCATACTGCACTCAGGCAAGCAATTCTTTGCTGGGGGGAACTAATGACTCTAGCTACCTGGGTGGGTGTTAATTTGGAAGATCCAGCGTCTAGAGACCTAGTAGTCAGTTATGTCAACACTAATATGGGCCTAAAGTTCAGGCAACTCTTGTGGTTTCACATTTCTTGTCTCACTTTTGGAAGAGAAACAGTTATAGAGTATTTGGTGTCTTTCGGAGTGTGGATTCGCACTCCTCCAGCTTATAGACCACCAAATGCCCCTATCCTATCAACACTTCCGGAGACTACTGTTGTTAGACGACGAGGCAGGGAACCTAGAAGAAGAGAACCCGAGCCTCGCAGACGAAGGGAACAAGAGCCGCGTCGCAGAAGAGAGCAAGAACGGGAACCTCAATGTTAGCTGCAGAAAA
