# Supplementary material for: Amino acid residues at core protein dimer-dimer interface modulate multiple steps of hepatitis B virus replication and HBeAg biogenesis
Source: PLoS Pathog. 2021 Nov 9;17(11):e1010057. doi: 10.1371/journal.ppat.1010057 (PMC8604296; doi:10.1371/journal.ppat.1010057)
Supplement: S4 Table — (DOCX) [file ppat.1010057.s014.docx]

**S4 Table. Information of antibodies used.**

| Antibody | Target | Company | Cat. NO. | Dilution | Assay |
| --- | --- | --- | --- | --- | --- |
| HBc170A rabbit polyclonal antibody | Core C-terminal 170-183aa | GenScript | Customized production | 1:1000 | Western blot |
| β−actin（8H10D10）mouse antibody | Human b-actin | CST | 3700 | 1:1000 | Western blot |
| HA-tag (C29F4）antibody | HA epitope | CST | 3724S | 1:1000 | Western blot |
| HA-tag (C29F4）antibody | HA epitope | CST | 3724S | 1:100 | IF |
| HBcAg（1-5）mouse antibody | capsids | Santa Cruz Biotechnology | sc-52406 | 1:200 | Particle gel assay |
| HBs（anti-Ad/Ay）horse polyclonal antibody | HBs | Abcam | ab9193 | - | IP-qPCR |
| PreS2 mouse monoclonal antibody | PreS2 | Abcam | Ab8635 | - | IP-qPCR |
| Calnexin（C5C9）rabbit polyclonal antibody | Human Calnexin | CST | 2679 | 1:1000 | Western blot |
| Lamin(A/C)（E-1）mouse antibody | Human Lamin(A/C) | Santa Cruz Biotechnology | Sc-376248 | 1:200 | Western blot |
| α/β-tubulin rabbit antibody | a/b-tubulin | CST | 2148 | 1:1000 | Western blot |
| Puromycin mouse monoclonal antibody | Puromycin | Sigma | MABE343 | 1:5000 | Western blot |
| HRP-linked mouse IgG secondary antibody | Mouse IgG | CST | 7076S | 1:5000 | Western blot |
| HRP-linked rabbit IgG secondary antibody | Rabbit IgG | CST | 7074S | 1:5000 | Western blot |
| Li-Cor IRDye goat anti rabbit secondary antibody | Rabbit IgG | Li-Cor | C90408-08 | 1:5000 | Western blot |
| Li-Cor IRDye goat anti mouse secondary antibody | Mouse IgG | Li-Cor | C81106-05 | 1:5000 | Western blot |
| Alexa Fluor 488 goat anti mouse IgG (H+L) secondary antibody | Mouse IgG | Invitrogen | A-11029 | 1:2000 | IF |
| Alexa Fluor 488 goat anti rabbit IgG (H+L) secondary antibody | Rabbit IgG | Invitrogen | A-11034 | 1:2000 | IF |
